# On Lightweight Shape Memory Vitrimer Composites

**DOI:** 10.1021/acsapm.3c01749

**Published:** 2023-12-15

**Authors:** Siavash Sarrafan, Guoqiang Li

**Affiliations:** Department of Mechanical & Industrial Engineering, Louisiana State University, Baton Rouge, Louisiana 70803, United States

**Keywords:** shape memory polymer, vitrimer, syntactic foam, hollow glass microspheres, lightweight

## Abstract

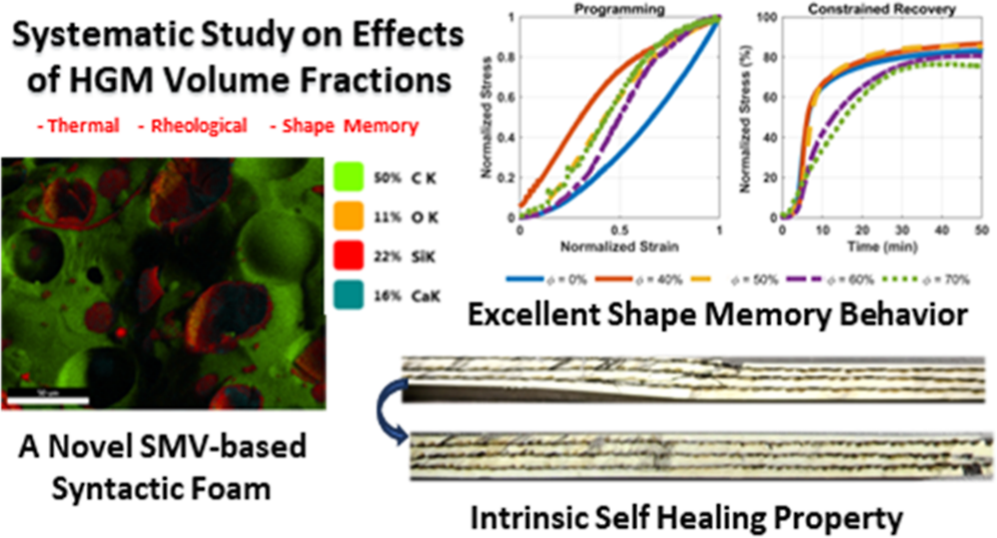

Lightweight materials are highly desired in many engineering applications. A popular approach to obtain lightweight polymers is to prepare polymeric syntactic foams by dispersing hollow particles, such as hollow glass microbubbles (HGMs), in a polymer matrix. Integrating shape memory vitrimers (SMVs) in fabricating these syntactic foams enhances their appeal due to the multifunctionality of SMVs. The SMV-based syntactic foams have many potential applications, including actuators, insulators, and sandwich cores. However, there is a knowledge gap in understanding the effect of the HGM volume fraction on different material properties and behaviors. In this study, we prepared an SMV-based syntactic foam to investigate the influence of the HGM volume fractions on a broad set of properties. Four sample groups, containing 40, 50, 60, and 70% HGMs by volume, were tested and compared to a control pure SMV group. A series of analyses and various chemical, physical, mechanical, thermal, rheological, and functional experiments were conducted to explore the feasibility of ultralight foams. Notably, the effect of HGM volume fractions on the rheological properties was methodically evaluated. The self-healing capability of the syntactic foam was also assessed for healing at low and high temperatures. This study proves the viability of manufacturing multifunctional ultralightweight SMV-based syntactic foams, which are instrumental for designing ultralightweight engineering structures and devices.

## 1 Introduction

Over the years, shape memory polymers (SMPs) have been a popular research topic for researchers in academia and industry.^1−5^ Their unique properties, which allow them to be used as actuators or conformable insulators controlled by various external triggers such as heat, light, electric current, and magnetic fields, have made them incredibly attractive for studies. One of the attractions of polymers, in general, is their low weight. Subsequently, SMPs, particularly as compared to shape memory alloys, are notably appealing for applications where low density is required or beneficial.^6,7^

Incorporating hollow bubbles of different sizes into the SMP is an excellent method for further reducing the density of the final material without significantly compromising its mechanical properties. Since the first SMP-based syntactic foam was reported by Li and John,^8^ many SMP-based syntactic foams have been studied, including SMP-based syntactic foams with extrinsic self-healing capabilities by incorporation of external healing agents,^9,10^ foam behavior under cyclic loading,^11^ and foam durability under environmental attacks.^12^ Constitutive modeling of SMP-based syntactic foams has also been conducted.^13−16^ Potential applications of SMP-based syntactic foams include using them as sandwich cores,^8^ deployable space structures,^17^ sealants in pavement and bridge deck joints,^18,19^ and loss circulation materials in the oil and gas and geothermal industries.^20^ Recent development includes SMP-based syntactic foams with multifunctionalities such as 3D printability, flame retardancy, strain sensing, and two-way shape memory effect.^21−23^

In addition to shape memory, damage healing in polymer composites is also a highly valuable functionality. Damage healing can occur either extrinsically by adding an external healing agent or intrinsically within the polymer itself. Although SMP-based syntactic foams with extrinsic self-healing have been investigated,^9,10^ none of the reported syntactic foams have intrinsic self-healing capability to date. It is clear that the intrinsic self-healing capability of syntactic foam is dependent on its polymer matrix. While intrinsic self-healing thermoset polymers have been a topic of intensive research over the years, vitrimer, a type of intrinsic self-healing thermoset polymer based on the adaptable covalent network (CAN), has become one of the most studied intrinsic self-healing polymers since the word of vitrimer was coined in 2011.^24^ Subsequently, many outstanding studies on vitrimers have been conducted.^25−32^ Several new reversible covalent bonds have been introduced in the thermoset network, including, but not limited to, transesterification,^33−35^ transcarbamoylation,^36^ transamination (to vinylogous urethane or hindered urea),^37^ olefin metathesis,^38^ siloxane equilibration,^39^ boronate-diol exchange,^40^ radical disulfide exchange,^41^ radical thiyl-ally sulfide exchange,^42^ and phosphate ester exchange.^43^ With the rapid development of intrinsic self-healing vitrimers, multifunctional vitrimers have been designed, synthesized, and tested, including vitrimers with shape memory effect,^44−50^ flame retardancy,^51,52^ and 3D/4D printability.^53−55^

From the literature survey, it is evident that many studies have focused on SMP-based syntactic foams. However, the effect of the volume fraction of the hollow particles on the shape memory effect and other properties is largely unknown because most studies used a fixed particle volume fraction, and most of the time, 40%.^8−23^ Furthermore, no studies have been conducted using shape memory vitrimer (SMV) as the matrix to prepare SMV-based syntactic foams. Therefore, two apparent knowledge gaps emerge: the absence of research on SMV-based syntactic foams and a minimal understanding of the effects of the HGM volume fraction on the mechanical and functional properties. Therefore, this study aims to explore the possibility of higher volume fractions of HGMs in the SMV matrix to create a very lightweight material with a shape memory effect and intrinsic self-healing capabilities. Based on the maximum volume fraction that theoretically can reach ∼74% in a face-centered crystal (FCC) structure with a single particle size, the volume fraction of the hollow glass microspheres (HGMs) in the prepared syntactic foams is selected to range from 40 to 70% in 10% intervals. An SMV developed in our lab, which is formed by cross-linking diglycidyl 1,2-cyclohexane-dicarboxylate (DCN) with a low molecular weight branched polyethylenimine (PEI), is selected as the matrix.^56^ This SMV is unique because it has self-healing capability at both room temperature due to its abundant hydrogen bonds and at temperature above its glass transition temperature due to a transesterification reaction. SMV samples without HGMs are also prepared as the control. The shape memory effect, mechanical properties, rheological properties, and damage healing capabilities of SMV-based syntactic foams are systematically studied. This study provides an understanding of the effect of HGM volume fraction on the mechanical and functional properties of syntactic foams in general and bridges the current knowledge gap of using SMV to prepare syntactic foams in particular.

## 2 Materials and Methods

### 2.1 Raw Materials

DCN with the linear formula C_14_H_20_O_6_ was purchased from TCI Chemicals (Tokyo, Japan). Branched PEI, with the linear formula H(NHCH_2_CH_2_)_*n*_NH_2_ and a low average molecular weight (*M*_W_ ∼800 by light scattering and *M*_n_ ∼600 by gel permeation chromatography), was purchased from Sigma-Aldrich (St. Louis, Missouri). The K15 hollow microbubbles with a true average density of 0.15 g/cm^3^ (0.13–0.17 g/cm^3^) made from soda-lime-borosilicate glass were purchased from 3 M (Saint Paul, MN). Their isostatic crush strength was 300 psi (approximately 2.06 MPa), and their median diameter was 60 μm.

### 2.2 Preparation

The DCN was first heated to 60 °C to lower its viscosity. Then, PEI, which was at room temperature, was added to the warm DCN in a 1:1 mass ratio and stirred carefully in an aluminum foil mold until a uniform mixture was obtained. For the syntactic foam, to minimize the possibility of entrapped air in the sample, DCN was added to the HGMs. Both ingredients were then heated together before mixing. For a typical scale of the experiments, an initial mass of 50 g of DCN was often used; however, the procedure could be scaled as necessary. After adding the PEI, the three ingredients were blended again until fully homogeneous. The required amount of the HGM was calculated based on the intended volume fraction of hollow spheres to the whole syntactic foam. Since the reaction between DCN and PEI is exothermic and results in air bubbles, the mixture was positioned on a large piece of ice for temperature stabilization, with mixing continuing until the creation of new bubbles ceased. Immediately after, it was gently poured into the desired molds and pressed to ensure that the created voids were minimal. They were left to cure at room temperature for at least 3 h. The curing process then continued through two more stages: first at 100 °C and then at 150 °C, with each lasting 2 h. Once they were cooled to ambient room temperature, the specimens were removed from their molds.

Laminated composites were prepared for damage-healing evaluations. A steel mold of 15.3 × 15.3 × 0.6 cm was used to prepare the laminated composites. A steel plate was fastened to the bottom of the mold frame, and a Teflon sheet was placed between them to facilitate demolding. Then, the first layer of syntactic foam was applied, followed by a 0.3 mm thick layer of plain-woven fabric 3K, 2 × 2 Twill weave carbon with 3K warp purchased from Fiber Glast (Brookville, OH). The syntactic foam used contained 40% HGMs by volume. This process proceeded until four syntactic foam layers and three plain-woven carbon fabrics were used. A roller was used to ensure the layers were even during laminating, and the syntactic foam penetrated the carbon fabric. On the top of the laminate, another Teflon sheet was placed, followed by another steel plate. Eight C-clamps pressed the pieces together tightly at a certain pressure to seal the mold. During preparation, the assembly was placed over ice to reduce bubble formation due to the exothermic polymer curing that happens even at low temperatures. The laminates underwent the same 3-step curing process as the pure polymer before cutting them into 2.54 cm wide strips.

### 2.3 Characterization and Testing

Density was determined by dividing the weight of several cuboids of approximately 90 × 25 × 7 mm by their volume for each sample type and taking the average of the results. The weight of the specimens was measured using an XS105 microbalance by Mettler Toledo (Columbus, OH).

An FEI Quanta 3D FEG dual-beam electron microscope (Hillsboro, Oregon) was used to capture the scanning electron microscopy (SEM) images. The accelerating voltage and working distances were 20 kV and 10–12 mm, respectively. To avoid noise due to static charge, small specimens were first coated with a thin layer of platinum using an EMS-550X Sputter Coater by Emitech SAS (Montigny-le-Bretonneux, France). The electron backscattered diffraction (EBSD) and energy dispersive spectroscopy (EDS) were performed using a Pegasus system made by EDAX (Mahwah, NJ) integrated into the focused ion beam (FIB) of the same SEM. Post-processing was conducted using the APEX software. A Spectrum Two Fourier-transform infrared spectroscopy (FTIR) spectrometer manufactured by PerkinElmer (Waltham, MA) was used in the range of 4000–400 cm^–1^ to characterize the chemical bonds in the samples of about 1 mm thick. For each test, three samples were tested five times to ensure the accuracy of the results. The measurements were taken with 32 scans at a resolution of 4 cm^–1^.

For the thermal analysis of the samples, a DSC 4000 calorimeter made by PerkinElmer (Waltham, MA) was used. Samples of approximately 14 mg were scanned from −50 to 150 °C at 10 °C/min. The tests were performed in a nitrogen environment with a 20 mL/min gas flow rate. Each test was repeated at least twice. Thermogravimetric analysis (TGA) was conducted using a TGA 550 instrument by TA Instruments (New Castle, DE). The recorded thermogram was recorded from room temperature to 800 °C at a rate of 10 °C/min with nitrogen as the ambient gas.

The rheological tests were conducted using a Q800 DMA instrument by TA Instruments (New Castle, DE). Films approximately 7 mm wide and 2.5 mm thick with an effective length of 11 mm were used under the multifrequency/strain test mode. The temperature ramped from −30 to 150 °C at a constant rate of 3 °C/min. The frequency was set to 1 Hz, and the amplitude was selected to be 20 μm. Capturing the storage modulus in the linear elastic range of the specimens was done by using a logarithmically increasing amplitude sweep from 0.05 to 5 μm at 0.1 Hz. The effective length of the films was increased to approximately 16 mm to minimize the applied strain further. Tests were performed at 100 °C, which is much above the glass transition temperature of all of the examined samples. However, to ensure that the tests were performed in the rubbery zone and that the frequency effect was minimal on the recorded data, tests were repeated at 105 and 110 °C. Similar results at all temperatures verified that the frequency was small enough and that the samples were indeed in the rubbery phase.

Frequency tests were also performed by using the DMA machine. Specimens underwent logarithmically increasing frequencies from 0.1–150 Hz with 5 values per decade. The test was repeated in 5 °C steps, a temperature range that started from −30 to −20 °C depending on the samples, and ended at 95 °C. The frequency sweep was started once the temperature was stabilized for at least 5 min to ensure uniform thermal distribution in specimens. All calculations for the time–temperature superposition (TTS), including the shift factors and the master curve generation, were done using the instrument’s companion software, Rheology Advantage.

The stress relaxation and creep tests were done similarly. A strain of 0.6–1% was applied for the stress relaxation, and a stress of 0.015 MPa was applied for the creep tests. In both tests, the film was displaced for 10 min and then allowed to recover for 15 min before the temperature was increased by 5 °C. The relaxation tests were performed from 25 to 100 °C, while the creep tests were between 20 and 110 °C in 5 °C intervals. The specimens were kept at each temperature for 10 min for equilibrium before loading. The TTS master curves were generated based on the same parameters obtained from the frequency tests.

The tensile and high-temperature compressive tests were conducted with an eXpert 2610 UTM by ADMET (Norwood, MA), equipped with an 8900 N load cell. The tensile test was conducted on dogbone specimens per the ASTM D638 IV standard made in silicone molds. The specimens were sanded to ensure that all surfaces were smooth. After tightening the clamps, samples were first unloaded at a 0.1 mm/min rate until the created compressive longitudinal load due to the lateral shrinkage at the two ends vanishes. A displacement rate of 0.2 mm/min (strain rate of approximately 0.025%/min) was then applied to the specimens, and the corresponding load was measured. The compression tests were done on cylindrical specimens with a diameter of 8 or 15 mm and a length of about 12 mm made using plastic syringes as molds. For the tests at room temperature, samples were first preloaded to 1 N at a displacement rate of 0.2 mm/min to ensure full contact. Then, a 0.2 mm/min displacement rate was applied for the actual test. The preloading segment was 0.5 N at a rate of 0.1 mm/min for high-temperature tests. Before starting the test, the temperature of the F-280DT ADMET environmental chamber was controlled by an Omron E5AC-T digital controller (Kyoto, Japan) for 1 h so that the fixtures and the specimen inside reached thermal equilibrium. Each test was repeated at least three times.

The programming and stress recovery tests were also conducted with the same setup. At the same temperature and with the same preloading segment as the high-temperature compression test, cylindrical samples with a roughly 8 mm diameter and 10 mm height were first compressed. They were then immediately cooled by using a fan. After unloading, the samples were measured again and placed in the chamber. Each piece was preloaded further to 0.5 N at a 0.1 mm/min rate before setting the temperature back to 60 °C. The constrained heating measured the increase in load due to the stress recovery in the samples elicited by the temperature increase. The room-temperature compression tests were conducted on an MTS Landmark instrument made by Instron (Norwood, MA) using a 100 kN transduce. The procedure used for this test was identical to the one for the high-temperature compression tests. The specimens used for this test were approximately 13 mm tall and had a circular cross-section with a diameter of about 15.5 mm.

Impact tests were performed using a Dynatup 8250 HV drop-weight impact tester made by Instron (Norwood, MA). The hemispherical crosshead tip with a diameter of 12.7 mm was released 10 cm above the clamped laminated composite specimens. The ASTM D3763-18 standard was followed for the tests using 5.19 lb (∼2.35 kg) crosshead weight. The impact-damaged laminate composites were then pressed between two steel plates to enable the syntactic foam matrix to heal. Three processes were examined for the healing of damaged laminates. The first experiment was done at room temperature for 72 h with a compressive stress of 3 MPa. Another approach was tested by pressing the impact-damaged specimens at 60 °C, which is in the glass transition zone, for 1 h under a 1 MPa load. Lastly, the third healing process was the initial room-temperature healing, as mentioned above, followed by a second healing phase at 150 °C, which enables retransesterification. This second step lasted 2 h with a compression stress of 1 MPa. The healing times and loads were selected based on the initial healing test results of the syntactic foam with 40% HGM by volume and the prepared laminates.

Using Teflon molds measuring 79 × 8 × 26 mm, samples were prepared for all tests, excluding the impact, tensile, and compression tests. All samples were subsequently cut to the desired shape by an iQ 228Cyclone 7 in. dry-cut tile saw with a diamond blade manufactured by IQ Power Tools (Perris, CA).

## 3 Results and Discussion

### 3.1 Theoretical and Practical Limits of Volume Fraction

The maximum volume fraction of particles in particulate composites is influenced by factors like particle shape, size, and size distribution or gradation. For monosized spherical particles, the maximum packing density is 74% for the face-centered cubic (FCC) arrangement. When particles possess specific size distributions, their packing density can increase. While theoretically the packing density can approach 100%, in practice, this is not achievable for particulate composites such as syntactic foams. The reason lies in the HGMs not adhering to the theoretical size distribution, preventing smaller particles from filling the open spaces among larger ones. To create an optimal composite, each particle should be coated with a thin polymer layer, preventing direct and dry contact between them. Given the polymer layer thickness, the particle volume fraction usually remains below 100%. Also, as the particle volume fraction rises, the composite’s viscosity increases, complicating the manufacturing process. Although adding a diluent can make manufacturing easier, too much diluent will negatively affect the mechanical and functional properties of the composites. Therefore, in this study, the maximum HGM volume fraction practically achieved was 70%. Further topics regarding the theoretical and practical limits of the volume fraction of HGMs in syntactic foams, including modeling limitations, various unit volumes, and random packing, are discussed at the end of the Supporting Information under Section S1. Additional details are discussed, such as the limitations specific to the polymer-based syntactic foams. These include the minimum volume fraction of the matrix, potential damages or deformations of the hollow bubbles, and size gradation or distribution of the added microspheres.

While there is no definite answer to what the maximum volume fraction could be for syntactic foams, this research aims to determine this limit empirically using the mentioned theoretical limits as guides. Apart from the manufacturing challenges of achieving the maximum volume fraction, this research also investigates at what point this volume fraction might significantly compromise the foam’s mechanical properties.

### 3.2 Porosity and Density

**Figure 1:**
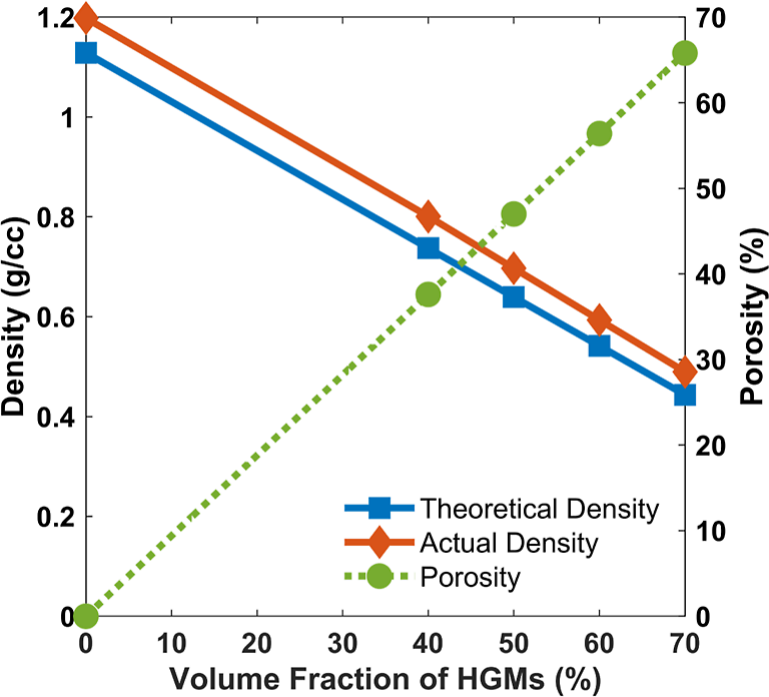
Comparison between theoretical and actual densities, alongside the porosities of the samples in relation to the volume fraction of the HGMs they contain.

The closed-cell porosity of syntactic foams due to the hollow glass bubbles can be calculated using eq 1
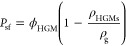
1where ρ_g_ is the density of the soda-lime-borosilicate glass and is considered to be 2.5 g/cm^3^. ρ_HGM_ is the density of the glass bubbles and was considered as 0.15 g/cm^3^. The change in porosity with the volume fraction of HGMs can be seen in Figure 1. The calculated porosities for the prepared samples can also be found in Table S1 in Supporting Information.

The theoretical density (ρ_th_) of the samples could be determined from the volume fraction of the HGMs utilized and the density of the constituents by eq 2
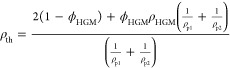
2where ρ_p1_ and ρ_p2_ are densities of the two constituents (DCN and PEI) and were equal to 1.22 and 1.05 g/cm^3^, respectively. ϕ_HGM_ is the volume fraction of the glass bubbles and varies from 0 to 70% in this study.

The theoretical density of each sample was calculated using eq 2 and compared to their actual measured density (ρ_a_) as plotted in Figure 1. The actual density for all samples is higher than the theoretical density. The observed increase in the actual density over the theoretical value is attributed to moisture absorption through the abundant hydrogen bonds in the DCN-PEI network.

As Figure 1 suggests, both densities follow a linear trend with respect to the volume fraction. In addition, the difference between the measured and calculated densities is very similar among different samples. This difference (ρ_a_ – ρ_th_) was calculated for each sample, and since it was perceived to be due to moisture absorbed from the environment, it was divided by the volume fraction (ϕ_p_ = *V*_p_/*V*_T_) and mass fraction of the polymer in each sample. The results in Table S1 reveal that the calculated  was quite minimal for all samples, suggesting the moisture absorption is probably linked to the mass of the polymer rather than its volume. However, further investigation into the effect of moisture on other properties, such as the constitutive behavior of the SMV-based syntactic foam, is beyond the scope of this study. Readers interested in this issue can refer to.^57,58^

### 3.3 Microstructure and Molecular Structure Characterization

**Figure 2:**
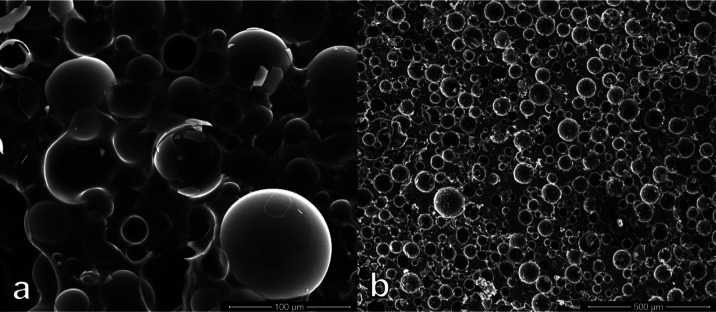
SEM image of the prepared syntactic foam with (a) 70 and (b) 40% HGMs by volume.

SEM was used to ensure that the microbubbles were dispersed uniformly inside the polymer matrix and to visually evaluate the syntactic foam’s quality. In the syntactic foam shown in Figure 2a, which contains 70% glass bubbles, some bubbles broke during preparation but the majority stayed intact. The high volume fraction of the HGMs is evident in this image, with many glass bubbles in close contact, surrounded by a relatively thin layer of polymer. As a point of reference, compare this to Figure 2b for only 40% HGMs by volume in which ample polymer matrix between the bubbles facilitates shape conformity through their viscoelastic properties.

Although this may not be clearly evident in the figures above, two more traits were observed by comparing the microstructure images. First, some bubbles were slightly deformed in the samples with Φ = 70% due to the applied pressure of the neighboring bubbles. This effect was not observed in syntactic foams with a lower volume fraction. Second, the prevalence of out-of-place broken pieces of glass bubbles was roughly 10–15% more per bubble in samples with 70% HGMs by volume than those with 40% HGMs. Typically, mixing the higher ratios of glass bubbles in polymer tends to be more time-consuming and requires significantly more effort. While it is impossible to categorically rule whether the bubble breakage was dominantly due to the increase in the frequency of their contact with the spatula or with the other bubbles, an increase in the volume fraction resulted in a more significant loss of HGMs. It must be mentioned that because the risk of damaging bubbles was higher, the syntactic foam samples with higher volume fractions were prepared much more carefully. Therefore, it is complicated to analyze the rate of bubble loss quantitatively. Please note that crushed bubbles with pieces still held together are probably due to the fracture on the surface prepared for the SEM specimen. There is a slim chance that those were broken during curing or cooling. However, the displaced pieces were most certainly broken during mixing, which is believed to be primarily responsible for damaging hollow bubbles and dispersing them throughout the sample.

**Figure 3:**
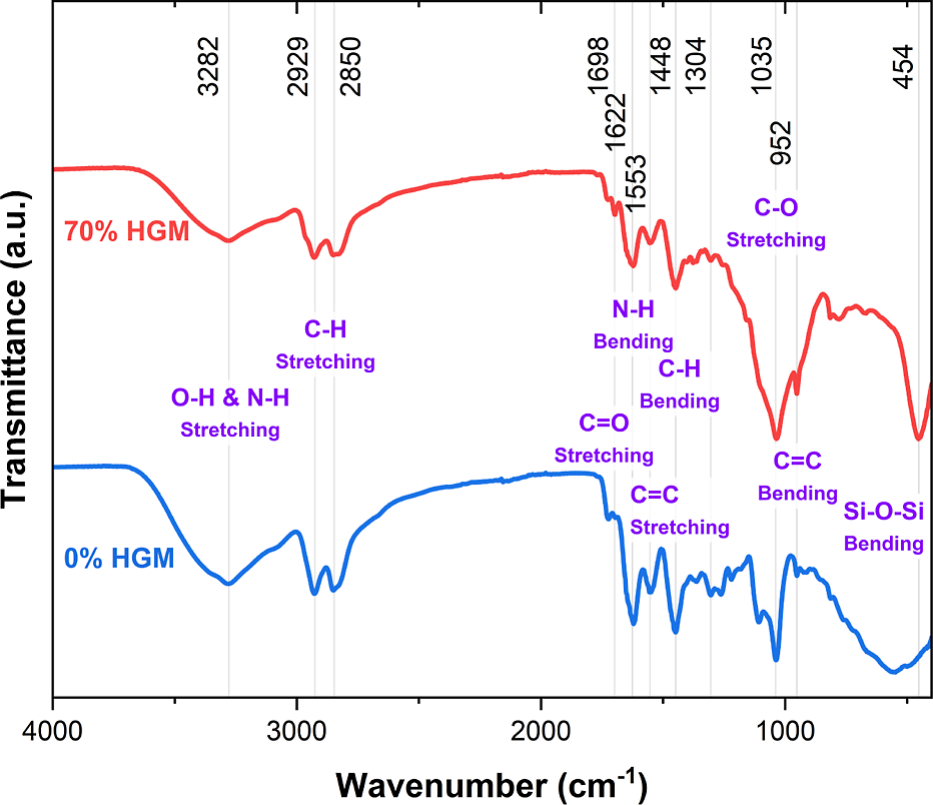
FTIR transmittance of the syntactic foam compared with the control polymer. The frequency of the peaks is marked with gray vertical lines, and their locations are noted above the figure. The predominant bonds attributed to the peaks are denoted in purple.

The effect of adding microspheres on the chemical structure of the syntactic foam was studied by using FTIR. As indicated by the gray vertical lines in Figure 3, the primary peaks of the spectrum remain consistent even after the addition of glass bubbles. The only exception is the added peak at around 454 cm^–1^ due to the silicon–oxygen bonds of the silicate glass spheres. As Figure 3 indicates, the broad peak at about 3282 cm^–1^ can be attributed to both the O–H bonds and the amine N–H bond stretching. The two peaks at 2929 and 2850 cm^–1^ are probably due to C–H bond stretching. The peaks observed at 1698, 1622, and 1553 cm^–1^ can be attributed to C=O stretching, N–H bending, and C=C stretching, respectively. C–O stretching and C=C bending are accountable for the peaks at 1035 and 952 cm^–1^, respectively. The FTIR spectra of all prepared samples are plotted and compared in Figure S1 in Supporting Information. Since the peaks are primarily unaffected by incorporating the HGMs, it can be concluded that they have not altered the chemical bonds within the DCN-PEI matrix, and the bonding between them is only physical.

**Figure 4:**
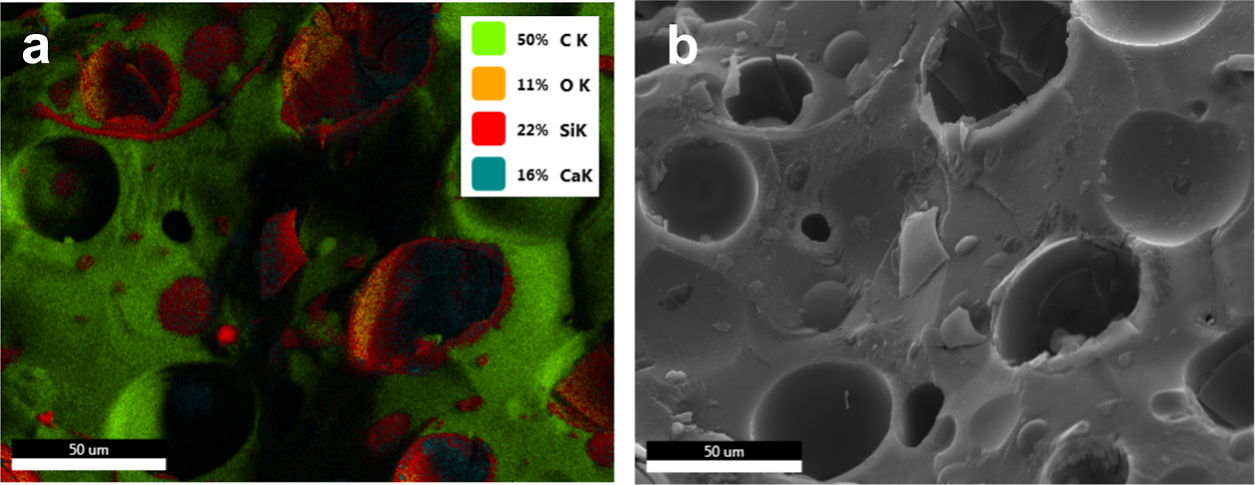
(a) Element overlay analysis done by EDS of the SEM image shown in (b) for the syntactic foam.

Figure 4a, which shows the surface element map of the SEM view in Figure 4b, clearly highlights the presence of silicon atoms in the broken glass pieces and on the surface of the hollow bubbles. Figure S2 in the Supporting Information shows the map for each of the elements separately. Note that limited by the detector’s resolution and the energy resolution of the electrons of the EDS, the X-ray energies of two or more elements are sometimes so close, or overlap, that they are considered one phase. Figure S3 in the Supporting Information is the phase map of the surface, which also considers some regions on the surface as unallocated parts. The energy intensities for each atom in these phases are shown in Figures S4–S8 in the Supporting Information. The ZAF-corrected EDS results estimating each element’s weight and atomic percentage for these phases are outlined in Tables S2 and S6 in the Supporting Information.

### 3.4 Thermal Behavior

**Figure 5:**
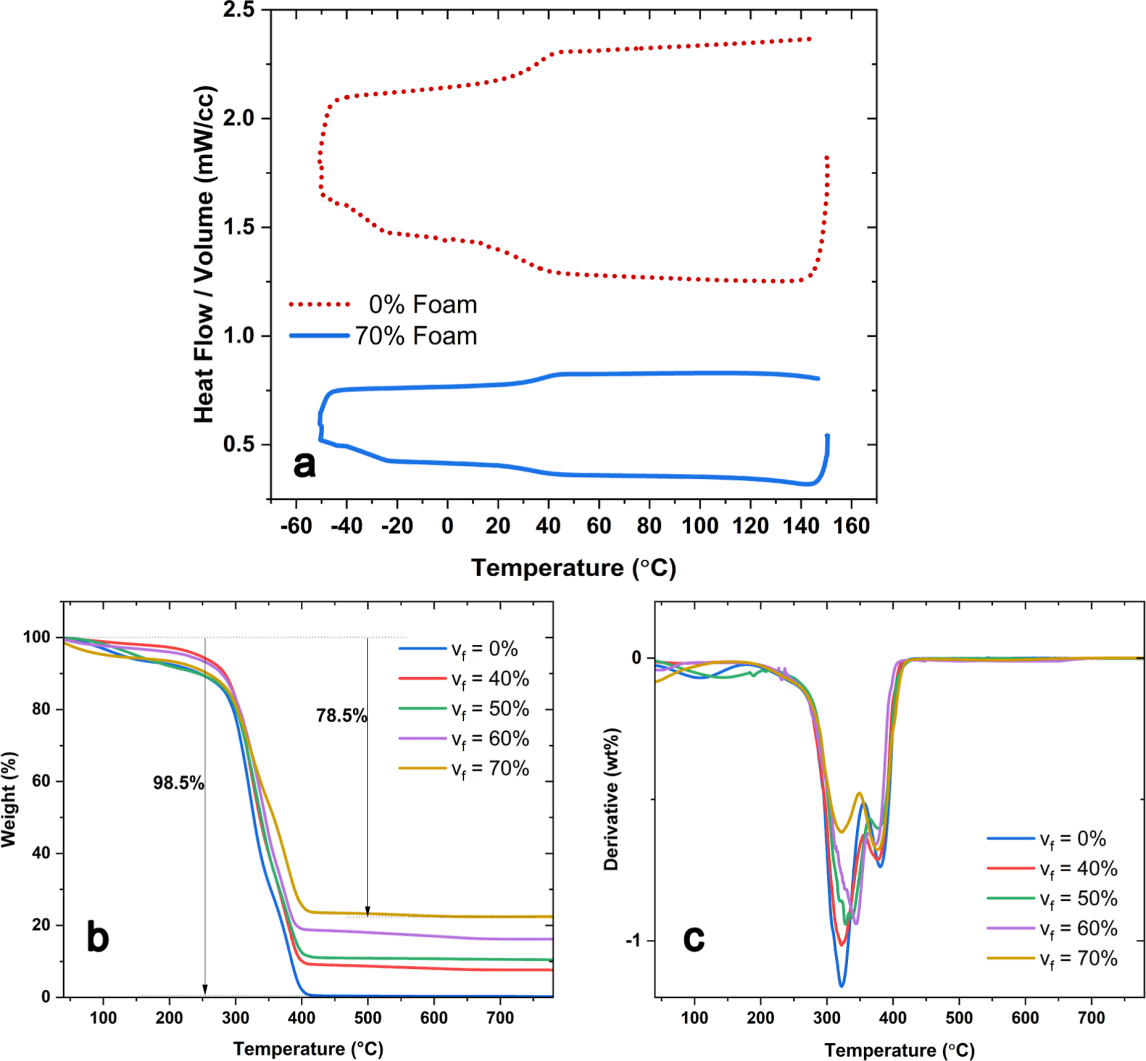
(a) DSC plots comparing the normalized heat flow of the syntactic foam with the pristine polymer matrix during the second cooling and heating segments. (b) TGA results showing the change in weight with temperature for samples with different volume fractions of HGMs and (c) derivative of the change in weight with temperature.

The DSC thermogram for the lightweight syntactic foam is compared to that for the pristine polymer in Figure 5. The syntactic foam containing 70% HGMs volume fraction is chosen for its highest bubble content. Only the second heating/cooling cycle is shown to eliminate any effects of the specimens’ thermal history. Since the hollow bubbles occupy a large portion of the syntactic foam’s volume, heat flow per unit volume of the foam is significantly less than the pure polymer. Using the midpoint of the extrapolated heat capacity method, the glass transition temperature (*T*_g_) was calculated as 32.52 °C for the pristine polymer and 36.68 °C for the syntactic foam. The increase in the glass transition temperature can be attributed to the presence of HGMs limiting the free volume of the polymer chains. As Figure S9 in the Supporting Information indicates, the *T*_g_ increases slightly with the increase in the volume fraction of the HGMs. This temperature was calculated as 34.62, 34.80, 35.53, and 36.88 °C for syntactic foams with 40, 50, 60, and 70% HGMs by volume, respectively.

The thermal stability of the prepared syntactic foams was examined and compared with that of the polymer with no glass bubbles inside. The change in weight of the samples with temperature is plotted in Figure 5b. In all instances, the weight slightly decreases at low temperatures, possibly due to the evaporation of the moisture they had absorbed. The primary decomposition initiated at approximately 300 °C, marking the onset of polymer matrix breakdown. At about 400 °C, almost all of the pure polymer sample was decomposed. The remainder weight of the specimens increases with an increase in the volume fraction of HGMs. It is therefore reasonable to assume that the HGMs are thermally stable within the syntactic foam samples. The remaining borosilicate glass in the analyzed syntactic foams resulted in a stable weight of the specimens up to 800 °C. However, the thermal stability of the glass does not imply that the bubbles retained their spherical shape. The derivative of the weight, plotted against temperature in Figure 5c, indicates that the polymer’s decomposition event happened in two stages. The monomer DCN and the cross-linker PEI used in this study are in a nonstoichiometric ratio, leading to abundant hydrogen bonds ascribed to the –NH, –OH, and C=O moieties in the thermoset DCN-PEI network. The lower hydrogen bonding energy leads to the first peak of the mass loss in the DCN-PEI network at about 300 °C. At about 400 °C, the covalent bonds break, leading to the second peak of the mass loss in Figure 5c. The initial stage of the thermogram, showing a slight weight loss of the polymer at and around 100 °C, corresponds approximately to a fraction similar to that calculated earlier for absorbed moisture. However, different samples showed different levels of weight loss in this temperature zone. This difference may be due to the storage environmental conditions, the duration of exposure to these conditions, and the microstructure of the samples, which might influence the moisture absorption and evaporation rates.

### 3.5 Rheological Properties

**Figure 6:**
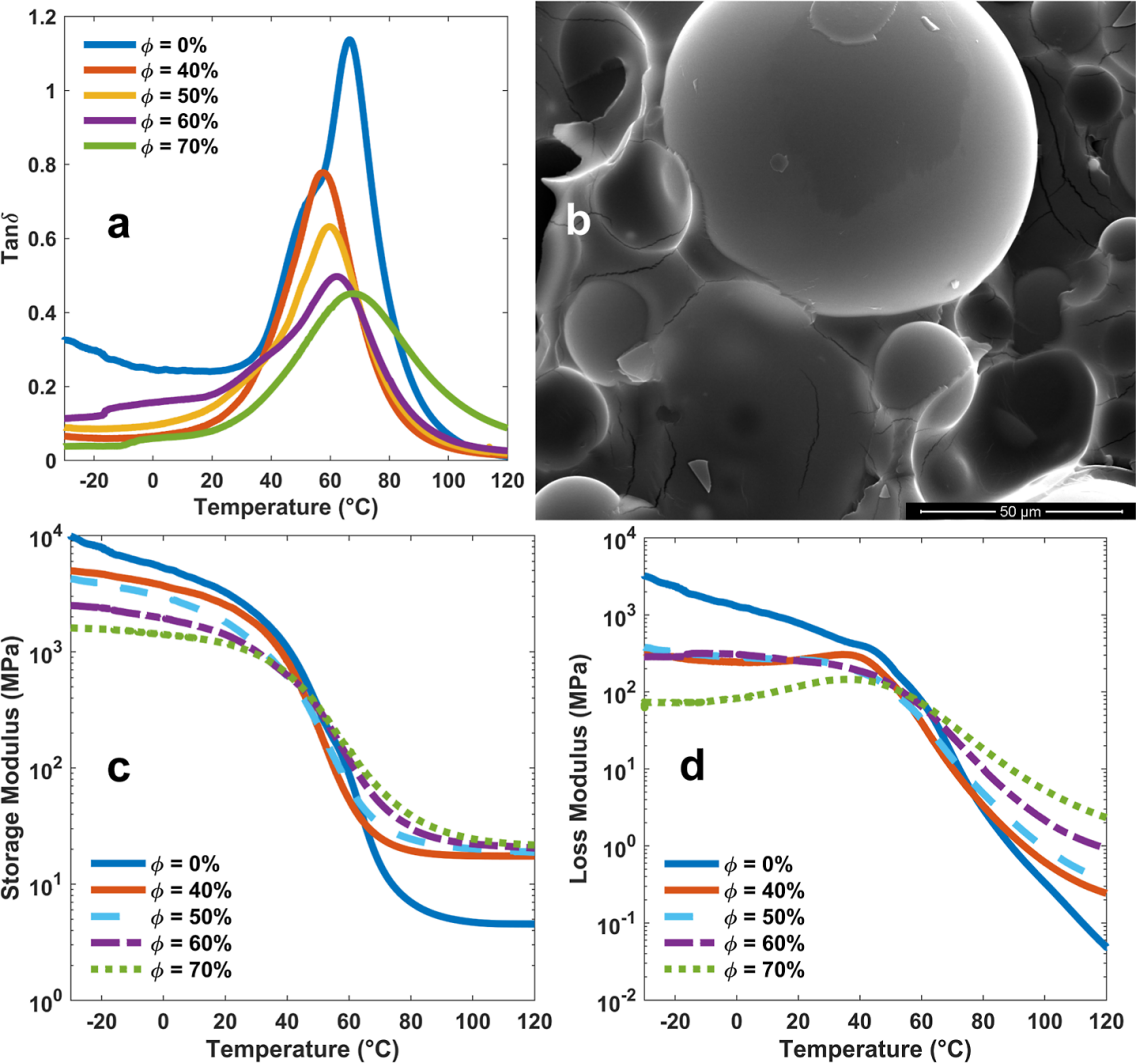
(a) Comparison of the tan δ peaks for the tested samples with different volume fractions of HGMs. (b) SEM image of a bubble at the fracture surface showing an almost complete debonding between the hollow glass bubble and the polymer matrix, (c) storage modulus variation with temperature, and (d) loss modulus variation with temperature.

The temperature scan of the samples prepared with different HGMs volume fractions performed by using DMA also showed a change in the glass transition temperature with the bubble contents, as depicted in Figure 6a. The *T*_g_, marked by the peak of the tan δ curves, shows a significant drop when 40% bubbles are added to the pure polymer. This drop is believed to result from the effect of the glass bubbles acting as barriers to prevent a larger intertwining polymer network through cross-links. Due to the interruption in forming a denser network, the thermal energy required for the motion of segments decreases. As a result, the glass transition temperature of the SMV-based syntactic foam is lowered.

However, with the increase in the HGM volume fractions, the glass transition temperature of the SMV-based syntactic foams started to increase. It is worth noting that HGMs also serve as mechanical barriers, inhibiting the movement of the chains. Therefore, two competing mechanisms existed in the SMV-based syntactic foams. On the one hand, with the incorporation of HGM particles, the SMV network formation was interrupted, reducing the glass transition temperature. On the other hand, with the increase in the HGM volume fraction, the hindrance to segmental rotation increases, leading to an increase in glass transition temperature. Moreover, the hollow bubbles have a thermal conductivity much smaller than that of their surrounding polymer matrix. Therefore, at identical heating rates, heat takes longer to reach the inner chains than to reach the inner chains close to the surface. Both of these factors are believed to contribute to the observed increase in *T*_g_ in the syntactic foam samples as the HGM volume fraction (Φ) rises.

Please note that segmental rotation and motion are complicated and affected by many factors. Although the DMA and the DSC are known to be slightly different in their sensitivity to the activities at different size scales, both instruments show a fairly wide range for the transition zone that is considerably overlapped. The observed discrepancy in the trend of *T*_g_ values between the two measurements and the theorized effects of HGMs should be interpreted in this context.

It must be mentioned that the observed effect of adding HGMs to a polymer matrix is not universal. Since their addition has multiple effects on the polymer network, as mentioned earlier, depending on which factor is dominant, the glass transition can be shifted in both directions. Therefore, their size, rigidity, and bonding strength between them and the matrix influence the final *T*_g_. The chemical adhesion between the bubbles and polymer can be investigated through different techniques, such as FTIR, as done above.

To some degree, the physical bonding between the two phases can also be studied by observing the interfacial transition zone (ITZ) between the bubbles and matrix after debonding. The high-magnification SEM images taken from the bubbles show only a tiny portion of the thin ITZ layer that remains on the bubbles after debonding (Figure 6b). When there is strong adhesion between the two bodies, the debonding mostly happens between the ITZ and the matrix. In contrast, weak bonds lead to ITZ peeling from the bubble surface. The relatively abrupt change in the chemical compositions near the hollow bubbles captured by EDS, as shown in Figure S10, can also be explained similarly. Here, traces of the chemical elements on the path illustrated in blue are determined and plotted. A similar analysis of a line drawn on the pure polymer is shown in Figure S11 for comparison. Further details on microstructural and rheological effects of the coalescence between fillers and polymer matrices are widely reported and discussed.^59,60^ Expectedly, high coalescence via hydrogen bonding increases the *T*_g_.

Figure 6c shows that the pristine polymer exhibits a higher storage modulus due to a more extended and robust network. The interrupted network and the lower stiffness of the hollow bubbles result in a lower storage modulus. As the polymer’s stiffness declines at higher temperatures, the storage modulus highly depends on the volume fraction of the HGMs that are more rigid than the rubbery matrix.

The effects of HGM volume fractions on the motion of polymer chains can also be observed by comparing the loss moduli, as plotted in Figure 6d. All tests performed on the pure polymer showed a constant decrease in the loss modulus at a relatively stable rate before the rate suddenly increased in the glass transition region. However, the initial decline in the loss modulus at low temperatures for all syntactic foams was followed by an increase. As expected, the loss modulus in the rubbery zone is dominated by the effect of these inclusions and monotonically increases with the HGM volume fraction. The storage and loss moduli of all samples at three temperatures of −20, 30, and 100 °C are reported in Table S7. These temperatures are selected for a better comparison of the effects of the HGM volume fraction in the frozen, room temperature, and rubbery states.

#### 3.5.1 Frequency Response

The rheological properties of three groups of samples (Φ = 0, 40, 60%) were also studied at different frequencies and temperatures (Figures S12–S14, respectively). The storage modulus increased with both the frequency and temperature in all three samples. However, at low temperatures, the rate in the pure samples was almost double that in the syntactic foams (i.e., the modulus at the highest frequency was 2.63 vs 1.40 and 1.22 times the modulus at the lowest frequency). In general, at low temperatures, the storage moduli of the syntactic foams were similar and much smaller than the pure polymer (e.g., 3245 vs 578 and 330.8 MPa at −5 °C and 150 Hz). Since the frozen polymer network was mainly responsible for the storage modulus at low temperatures, the differences can be attributed to the difference in the stiffness of the uninterrupted vs interrupted networks. As the frequency increased, polymer chains had less time to relax and come in contact with the glass bubbles. The strong covalent bonds in the polymer network and small segmental rotations primarily generated resistance to the applied loads in this situation. As a result, the storage modulus in the pure polymer understandably shows a considerably larger increase with frequency in comparison to the syntactic foams. For example, around their glass transition temperatures, the storage modulus of the pure SMV increased up to about 30 times with increasing frequency, while this number was about 13 for Φ = 40% and only 7 for Φ = 60%.

The influence of the frequency on different samples at glassy and rubbery states also provides insights into the microstructure of the composites. As shown in Figure S15, the loss modulus in the rubbery state (i.e., 95 °C) monotonically increased with frequency for all samples. However, there was a slight decline before the increase in the glassy state (i.e., −5 °C). Also note that when the frozen network was dominantly responsible for the loss modulus, the loss modulus decreased with the volume fraction of HGMs. This trend was entirely reversed once the glass bubbles were dominant in dissipating energy during deformation in the rubbery state.

The frequency response of the samples at different temperatures was used for time–temperature superposition analysis. The shift parameters and the fitted Williams–Landel–Ferry (WLF) model are plotted in Figures S16–S18, respectively. Since the shift factors for *E*′ and *E*″ were almost identical, the samples were assumed to be rheologically simple for the TTS calculations. To further ensure this, the smooth Cole–Cole plots are also drawn in Figures S19–S21, respectively. The resulting master curves are plotted in Figures S22 and S24. It is worth noting that the activation energy was also estimated for the samples using an Arrhenius fit to these data. The results show that the activation energy of the pure polymer decreased from 341.0 to 297.9 kJ/mol when a syntactic foam with 40% HGMs was compared. However, it again increased to 365.5 kJ/mol for the 60% HGM syntactic foam. The change in the activation energy can also be used as another indication of the mentioned effects of HGMs on the microstructure and the mobility of polymer chains.

In Figure 6a, the peak of tan δ, which usually corresponds to good damping properties, is the largest for pure polymer and decreases as the volume fraction of the HGMs increases. This is understandable, because the damping is a result of viscoelasticity. The HGMs are elastic materials that do not contribute to damping. However, the results of time–temperature superposition of performed frequency tests show that the syntactic foams may be more favorable to use at higher frequencies. For example, comparing the generated master curves at room temperature (30 °C) resulting from the frequency tests shows a higher peak for frequencies in the lower bounds of the hearing range (for example, 100 Hz) for the syntactic foam with Φ = 40% (Figure S23), than for the pure polymer (Figure S22). However, the loss factor again decreases at higher volume fractions (Figure S24).

#### 3.5.2 Cross-Link Density

The rheological changes in particulate-filled composites are often attributed to only the ITZ between the polymer matrix and the particulates.^61^ They are usually considered to be caused by local constraints. Therefore, their intensity strongly depends on the spacings between the particles.^62,63^ However, it is reasonable to believe they also impede the formation of certain cross-links within the polymer network, which could have potentially enhanced its integration and rigidity. The significance of this effect was evaluated by calculating the difference between the cross-linking density of pure polymer and the syntactic foams. Young’s modulus at the rubbery state was used to determine network integration, and since both matrix and particles affect the mechanical properties, their effects were decoupled.^64^ To estimate the modulus of the SMV matrix in the syntactic foams in the rubbery state, a model proposed earlier to predict the elastic properties of the syntactic foams with hollow bubbles was used to predict the modulus of the SMV matrix.^65^

Using the measured Young’s modulus of the SMV matrix in the rubbery state, which is 4.9 MPa, the modulus of the syntactic foams with 40 and 60% HGM per volume can be estimated as *E*_40_ = 4.61 MPa and *E*_60_ = 1.99 MPa. Here, *E*_40_ and *E*_60_ are Young’s moduli of the SMV matrix for the syntactic foams with Φ = 40 and 60%, respectively. Please note that since the polymer matrix is in the rubbery zone at this temperature, it can be assumed incompressible. Therefore, Poisson’s ratio, ν, can be assumed to be approximately 0.5.

The cross-link density of a polymer network (ξ_c_) can be estimated from the Young’s modulus (*E*) and temperature (*T*), using the Payne equation^66^ as
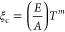
3where *A* and *m* are material-dependent constants. However, considering all parameters are the same in these three tests, the cross-link densities of the vitrimer networks are directly proportional to their moduli. This means dispersing the HGMs with a volume fraction of 40% only slightly decreased the cross-link density by approximately 6%. However, increasing the volume fraction of the HGMs to 60% significantly disrupted the interconnected network, reducing its cross-link density to only about 40% of the pure SMV.

### 3.6 Stress Relaxation Behavior

The relaxation modulus of a polymer is influenced by steric hindrance, which can be related to the reptation model of polymer dynamics.^67^ According to the reptation model, the relaxation of a polymer network is dominated by the motion of a single polymer chain as it is reptates through the network of entangled chains. The relaxation modulus is then related to the reptation time, which is the time it takes for the chain to move a distance equivalent to its contour length through the entangled network.^68^

The reptation time is influenced by various factors, including the length and stiffness of the polymer chain, the degree of entanglement in the network, and the steric hindrance of the chains.^69^ Steric hindrance, in particular, can significantly affect the reptation time by limiting the chain’s ability to move through the network. Chains with larger side groups or more complex branching structures will experience greater steric hindrance and slower reptation times, leading to lower relaxation moduli.^70^ Therefore, the relationship between a polymer’s relaxation modulus and its chains’ steric hindrance can be used to predict the mechanical properties of polymers with different chain structures or to design polymers with specific mechanical properties.

A series of relaxation tests was performed on the prepared syntactic foams with two different bubble volume fractions (40 and 60%) to compare their results with the pure polymer. Figure S25 shows an example of these tests running from 25 to 100 °C at intervals of 5 °C for the syntactic foam with Φ = 60%. The wide temperature range was selected for the TTS analysis, intending to encompass the samples’ glass transition temperature. To better understand the different mechanisms in the glassy and rubbery states, the glass transition temperature was also chosen as the reference temperature for generating the master curves. Therefore, the reference temperature for the syntactic foam samples with 40 and 60% HGM by volume was selected as 60 °C. For the pure polymer, the master curve was produced at 65 °C. However, to ensure the change in the reference temperature did not affect the analyses, a second master curve for the pure polymer at 60 °C was also calculated. It must be noted that the shift factors for the TTS analysis were based on the frequency TTS calculations presented earlier.

A discrete relaxation model (DRM) was fitted to the master curves to quantify the relaxation behavior. The employed DRM was based on a simple Maxwell material for viscoelasticity. From this model, the discrete relaxation modulus can be mathematically written as
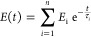
4where *E*_i_ is the initial relaxation modulus associated with each relaxation time τ_*i*_. The sum can be taken over all of the relaxation times in a system. But, limited by the software, only the first six primary spectra are studied here.

**Figure 7:**
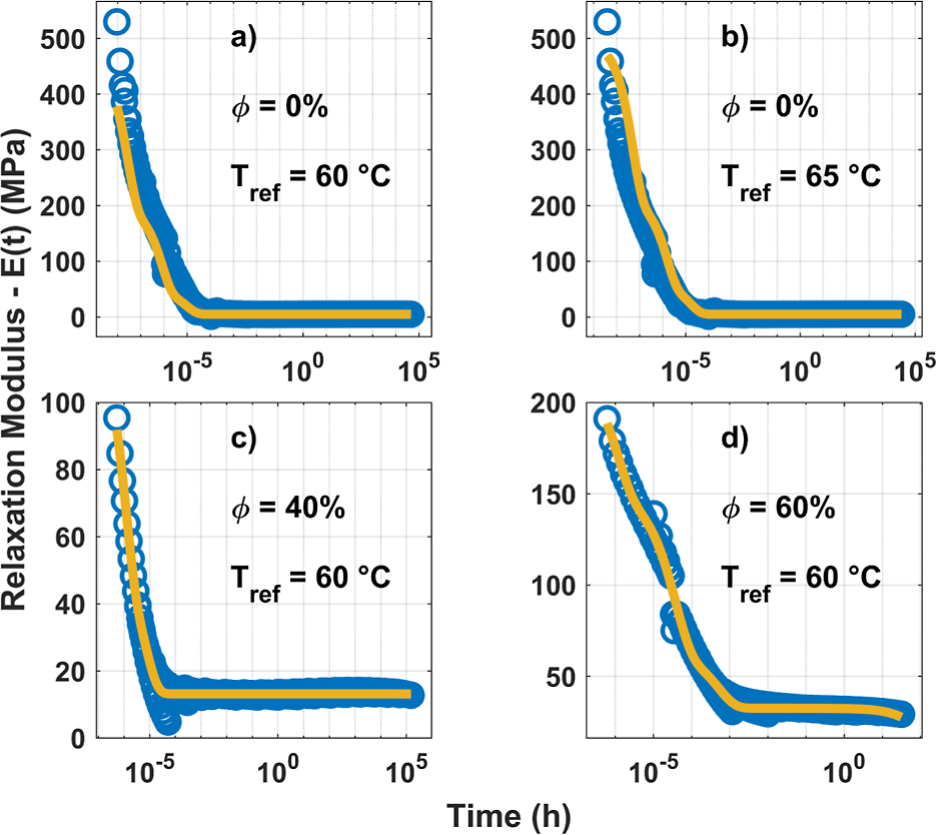
Master curves generated using the TTS analysis near the glass transition temperature for the relaxation modulus of the pure SMV in (a,b) and the syntactic foams with 40 and 60% HGMs by volume, respectively, in (c,d). The data points are shown with blue circles, and the fitted DRS model is in yellow. The horizontal time axis is on the logarithmic scale. The temperatures for which the master curves are calculated, which were also the same as the reference temperature for those calculations, are shown in each plot.

The calculated master curves for the pristine polymer at 60 and 65 °C are plotted in Figure 7a,b, respectively. Results for the syntactic foams are shown in Figure 7c,d. The calculated relaxation moduli for each time are shown with blue circles. The fitted DRM models on the curves are plotted in yellow. In all samples, the relaxation modulus is observed to stabilize with time.

The fitted DRM parameters and the final relaxation modulus, which was the last data point in the plots, for the analyzed samples are reported in Table S8. The lasting and most substantial response, denoted by *E*_1_, increased with the added particles’ volume fraction. This response can be attributed to the impediment the glass bubbles create for the continued motion of the polymer network. The effect of the HGM obstacles is also reflected in the final relaxation modulus, labeled as *E*_*t*→∞_ in Table S8. However, the spectra with lesser significance, E4, E3, and E2, in that order, follow an almost opposite trend. These responses are believed to be related to the cohesion of the polymer network, which sustains its integrity against the viscous motion that relaxes applied stress. As previously discussed, the HGM particles were believed to have prevented the formation of some cross-links. Therefore, they are speculated to decrease the integrity of the polymer matrix network and affect its response during stress relaxation. A similar trend can also be observed by comparing the relaxation of the three samples during a single cycle at two temperatures much below and much above the glass transition (Figure S26). In the frozen state (40 °C), the pure matrix with an unperturbed network had a considerably greater relaxation modulus, which shows its strong resistance to displacement. However, after the network conformed to the applied strain during the first 10 min of loading, it was more reluctant to return to its initial configuration in the subsequent 15 min recovery compared to the other two samples. The trend in the frozen phase was also dominated by the degree of disruption as the response order followed the HGMs’ volume fraction. Conversely, at higher temperatures (90 °C), where the SMV matrix was in the rubbery state and could flow, the two competing effects of the HGMs can be noticed. At Φ = 40%, the network cohesion was not afflicted too much by the presence of bubbles. Instead, they act as pinning points, complicating the rearrangement of the network. They also resulted in the network recoiling more and faster to its initial position. However, when the volume fraction increased too much, as for the sample with Φ = 60%, the interconnectivity of the network diminished to the extent that the relaxation modulus and the strain recovery both declined considerably, even compared to the pristine SMV. Please note that for the tests involving rheological properties, *T*_g_ was determined based on the DMA results.

### 3.7 Creep Behavior

The competing effects of the HGMs on the viscoelastic behavior of the syntactic foam were also observed in the results of the creep tests. Similar to the stress relaxation tests, experiments were performed at a series of temperatures, as shown in Figure S27. The creep compliance of the samples with the same three volume fractions of HGM (0, 40, and 60%) is compared with each other in Figure S28. At a temperature much below the *T*_g_ (20 °C), the pristine SMV with the highest network cross-link density had the slowest reaction to the applied load. The equilibrium creep compliance was also the largest for the pure SMV at this temperature, decreasing with increased Φ. As the network cohesion declined at temperatures above the *T*_g_ (95 °C) due to broken hydrogen bonds, the nonviscous glass in the syntactic foams substantially enlarged the difference between the pristine SMV and the foams’ behaviors. A similar trend was observed in the unloading of the samples. The integrity of the SMV matrix dominated the recovery speed at lower temperature. However, the unloading results at 95 °C showed that the syntactic foam with Φ = 40% had the fastest recovery response compared to the other two samples. This is again believed to be connected to the two opposing effects of HGMs in the polymer network. They act as barriers preventing the network segments from rotating or significantly rearranging. However, at very high volume fractions, they could impede the formation of the cross-linked network, resulting in a less restricted motion. As expected, the pure SMV relaxed more slowly than both syntactic foams at this temperature.

Results of the tests at different temperatures were also compiled for TTS analysis. The generated master curves are plotted in Figure S29 for the three volume fractions at two temperatures (one below and one above the *T*_g_). Similar to the discrete relaxation model discussed earlier, a discrete creep spectrum model was used to fit each master curve. Likewise, it was written as
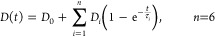
5Here, *D*(*t*) is the creep compliance of the material. *D*_*i*_ and τ_*i*_ are, respectively, the compliance and the time constant of the *i*th response spectrum. *D*_0_ is the initial response of the material. The calculated spectra for each sample at each of the two temperatures, with one in the glassy zone and the other in the rubbery zone, are listed in Table S9. The calculated viscosity (η_0_) and equilibrium response (*D*_*t*→∞_) are also reported in Table S9. The effect of the HGMs on preventing the segmental motion of the polymer network may again be evaluated through the most dominant compliances (i.e., *D*_1_ and *D*_*t*→∞_).

The pure SMV exhibited higher *D*_1_ and *D*_*t*→∞_ values compared to the syntactic foam samples. The difference was particularly pronounced at elevated temperatures, where the polymer network in its rubbery state readily conforms to the applied load. The dispersed HGMs appeared to impede the polymer’s mobility, slowing its creep motion under the applied load. However, despite the increase in the volume fraction of the HGMs from 40 to 60%, the creep compliance of the syntactic foam experienced a slight reduction instead of the expected increase. It is hypothesized that the pinning effect of the bubbles was overshadowed by the impact on the less integrated polymer network. In other words, the disruption caused by the bubbles impeding the formation of additional cross-links counteracted their anticipated influence on maintaining the network’s integrity. These results, along with the findings obtained for the stress relaxation and other rheological behaviors of the syntactic foams, demonstrate that the optimal behavior of the foams does not lie at the extremes of the volume fraction. Therefore, careful design of the HGMs’ volume fraction is necessary.

### 3.8 Mechanical Behavior under Monotonic Tension and Compression

#### 3.8.1 Tensile Test at Room Temperature

**Figure 8:**
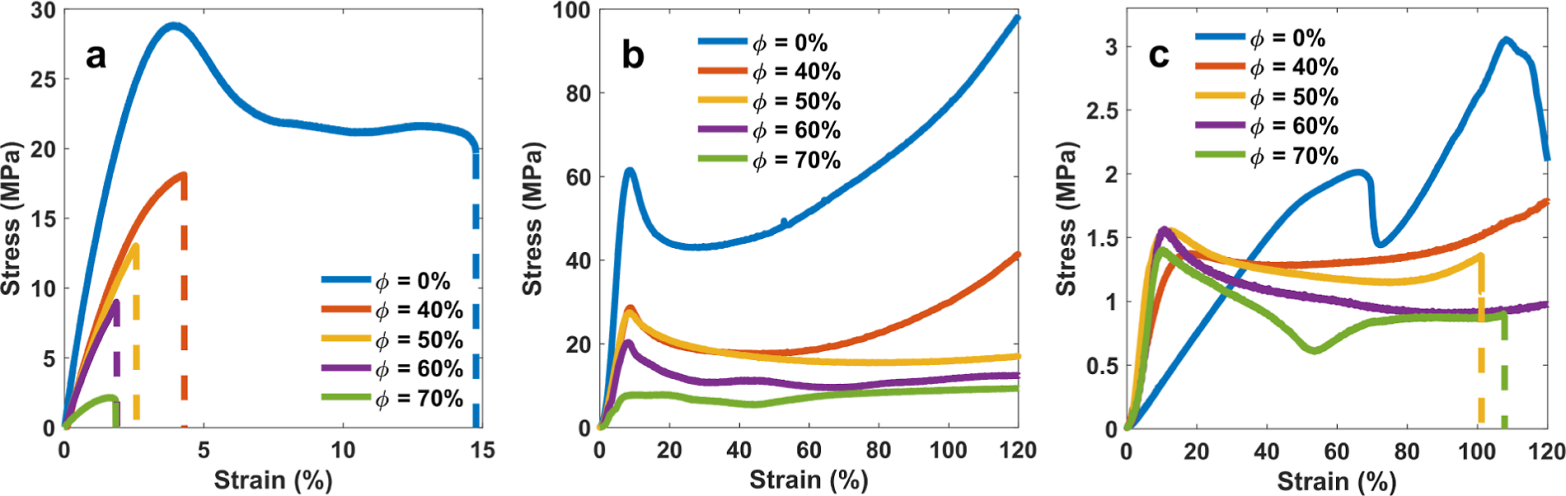
Results for (a) tensile test at room temperature, (b) compression at room temperature, and (c) compression at 60 °C (rubbery zone) for specimens with different volume fractions of hollow glass microbubbles. The dashed lines mark the failure of the specimen, which is ruptured under tension and crushing in compression. Plotted values are the true stress vs true strain.

The tensile test results for the different specimens are plotted in Figure 8a. The pure SMV shows the highest strength and elongation at break, which is believed to be due to its integrated cross-linked network. As the bonding among the polymer chains is interrupted by the HGMs, the strength and maximum elongation of the specimens decrease with the increase in the volume fraction of the bubbles. With the highest tensile strength of 28.9 MPa for the pure SMV, an increase in the HGM volume fraction changes the strength moderately to 18.1, 13.1, and 9 MPa for HGM volume fractions of 40, 50, and 60%, respectively, before another significant drop for Φ = 70% to 2.1 MPa.

The reduced bonds between the polymer’s cross-linked network also affect the modulus. As the tensile loading begins, the pure SMV shows the highest slope compared with all syntactic foams. The tangent line becomes less steep with an increase in the HGM volume fraction. Like strength, the modulus reduces substantially after introducing the HGMs but does not change considerably for the 40–60% volume fractions. However, there is a sharp decline in the modulus for Φ = 70%. This decline suggests that reaching a critical volume fraction will significantly decrease the mechanical properties. It is believed that once the network formation is interrupted by the glass bubbles, the fracture does not occur due to the breakage in the straightened segments but rather due to the debonding between the glass bubbles and the polymeric matrix. This happens due to the stress concentration at the HGM/SMV interface, which is reminiscent of composites made of a matrix stiffer than the particles. The elongation at the break of the specimens does not change considerably after a drop, once the glass bubbles are added. The tensile test for pure SMV also shows a distinctive softening region when the load reaches its maximum. This phenomenon in thermosets under tension usually marks the breakage of the cross-links between neighboring chains, including hydrogen bonds. Once most of these bonds are broken, the large chains straighten and slide against each other, leading to plastic flow and a plateau in the stress–strain curve. Since the presence of the HGM inclusions considerably reduces the formation of the network, this softening phase is not observed for the syntactic foams. Therefore, the maximum stress occurs almost immediately before rupture.

Strength, elongation at break, strain at maximum stress, and initial modulus for all specimens are summarized in Table S9. The initial moduli are calculated from the slope of a line at 0.2% strain tangent to a polynomial of sixth degrees fitted to each curve. The least-squares and the Vandermonde matrix methods are used to find the coefficients of the polynomials. Please note that the results plotted in Figure 8a are the true stress and strain values to account for the cross-sectional change during the relatively large displacement applied. However, the results in Table S10 are the more practical engineering stresses and strains for design. The modulus is also calculated from the engineering stress vs strain curve.

A comparison of the fracture surface of the specimens in Figure S30 also suggests that the failure mechanisms in the syntactic foams are different from those in the pure polymer. Figure S31 shows a flat surface, which means the rupture in the pure SMV was initiated by a brittle fracture, as expected. This crack propagated quickly throughout the material, leaving a shiny surface behind. On the contrary, although the syntactic foams’ fractures were also brittle and on a flat surface, it is slightly fibrous. This difference is because the failure is mainly divided into the debonding between the matrix and glass bubbles and the rupture of the polymer matrix in between those HGMs.

#### 3.8.2 Compression Test at Room Temperature

Due to their complex microstructure, the behavior of polymers is not identical in tension and compression. Adding the HMGs and the bonding between the two materials further increase this complexity. However, results from the compression tests plotted in Figure 8b suggest that a similar trend regarding the strength and modulus applies here. A distinguishable difference between the two tests is a softening stage, followed by a hardening stage in all specimens. This is typical for thermoset polymers under compression in the glassy state. The required strain and stress to overcome this barrier, however, depend on the integrity of the polymeric matrix, which reduces with an increase in Φ.

Remarkably, the stiffness of the syntactic foams shows no significant degradation with an increase in the volume fraction from 40 to 60%. The yield strength of the syntactic foam during compression is also nearly unaffected after increasing Φ from 40 to 50%. This can be seen in more detail in Figure S32, which shows only the initial portion of the results. The compression test behavior of the 70% syntactic foam again indicates the trade-off between the mechanical properties and the foam density at this fraction. The main mechanical properties of the specimens that resulted from the compression tests are summarized in Table S11. Like the tensile test, the true stress vs true strain is plotted to portray the behavior of the materials better as the cross-sectional area increases, particularly the softening phase after the stress peak. However, the results in Table S11 are based on the engineering stress–strain data. Please note that there is no maximum strain listed in the following table due to the absence of rupture in the compression test. However, contortions in the smooth stress plot suggest the failure onset as a fracture in the specimens. The method used to estimate the modulus in compression is identical with the one used in tension. However, due to the significant effect of geometrical imperfections of the two end surfaces of the specimens on the initial phase of stress–strain data, the modulus is here calculated at a higher strain, equal to 2%.

Specimens with a lower volume fraction of glass bubbles, including the pure SMV, underwent compression without a significant fracture being seen in them. These specimens are shown in Figures S33 and S34 after springback. However, the specimens with higher HGM volume fractions experienced longitudinal cracks on the outer surface. These cracks were vertical near the two ends and slanted in the middle. The fracture lines are visible in Figure S34.

#### 3.8.3 Compression Test in Rubbery Zone

The mechanical properties of the syntactic foam in its rubbery state are valuable for two main reasons. First, it is essential to know the maximum stress and strain that can be applied to the material during programming, which occurs in this state. Second, since the elastic and viscoelastic properties of the polymer matrix change substantially at the rubbery zone, the stiffness difference between the stiffer matrix and relatively softer inclusions reduces. Moreover, the decrease in the viscosity of the polymer allows the matrix to conform better to a shape change. Figure 8c shows the true stress vs true strain results for the different syntactic foams and the control pure SMV. As expected, the low stiffness of the SMV in the rubbery state decreases the total stiffness of the samples. Since the HGMs are more rigid than the polymer in the rubbery state, the stiffness of the syntactic foams slightly increases with Φ. However, the maximum strain at which the true stress reaches a maximum decreases with the increase in porosity. This change can be attributed to the increased chance of debonding, the elevated localized stress resulting from stress concentration, and the crushing of the HGMs. It is seen that the pure SMV displays two distinct sudden drops in the true stress–true strain curves. At 60 °C, the SMV network is in the rubbery state, implying that stress should rise monotonically with strain until fracture occurs. Therefore, the second drop in stress can be attributed to network fracture, which can be more clearly visualized in Figure S36 when viewed in terms of engineering stress and strain. The first abrupt drop in stress, unique to pure SMV, is believed to be due to the cleavage of the hydrogen bonds within its network. It is also observed that the SMV-based syntactic foams yield significantly lower strains, likely due to strain concentrations around the HGMs. This results in earlier cleavage of the hydrogen bonds at smaller test strains. The peaks in the stress–strain curves indicated the onset of a softening phase in the specimens that led to energy dissipation. However, significant stress drops were observed to result from crack formation, releasing strain energy. Samples with smaller drops usually showed no visible cracks. At the same time, the more significant reductions were generally accompanied by visible cracks or complete fractures and fragmentation. Figure S35 shows that the syntactic foam in its rubbery state broke at a 45° angle, cutting the sample into two-halves.

### 3.9 Shape Memory Behavior

The shape memory properties of the prepared syntactic foams and the effect of volume fraction on these properties are evaluated by their recovery stress during a constrained shape recovery test. Samples were programmed and recovered at 60 °C. This temperature was selected because it was believed to be in the rubbery state based on the DSC test results. The results obtained from the compression behavior in the rubbery zone (as seen in Figure 8c) were used to determine the programming strain. Repeated tests for different programming strains revealed that all samples, independent of their volume fraction, had a similar recovery stress efficiency of approximately 80% (±8%) as long as they did not undergo failure during programming. This efficiency is calculated as the maximum stress during recovery compared with the maximum stress during programming.

**Figure 9:**
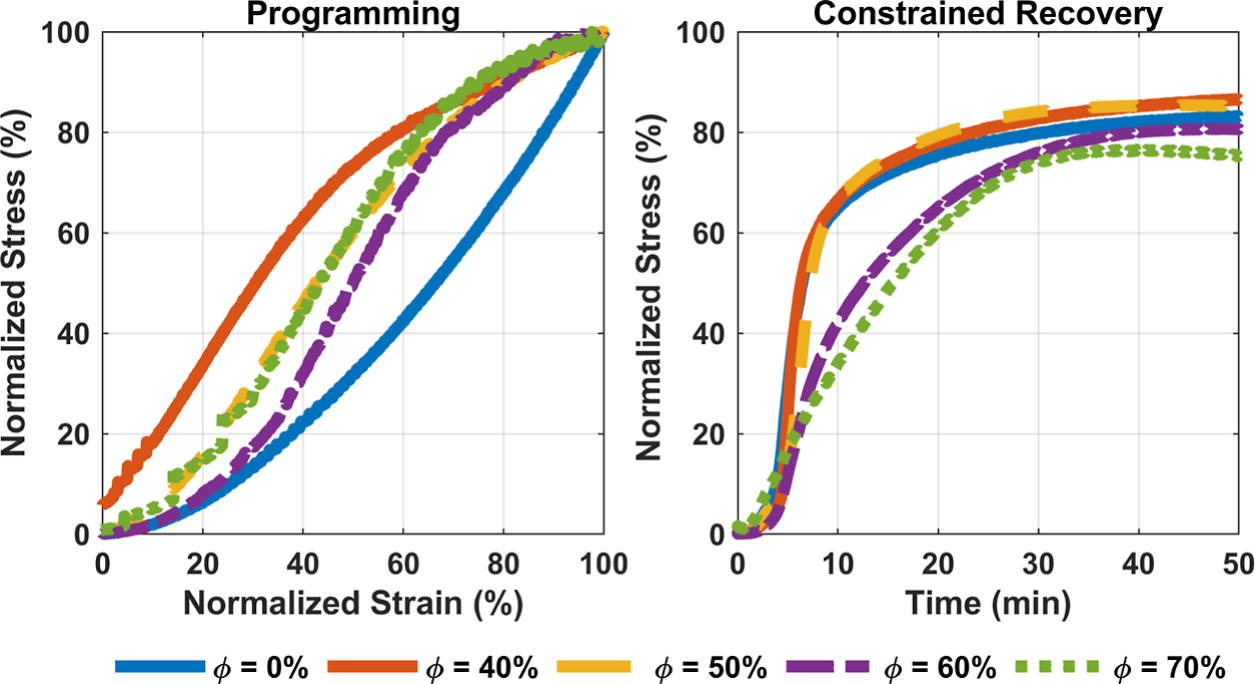
Programming and recovery stress for the prepared samples with different volume fractions of HGMs. All stresses and strains are engineering values. The stress values for each sample are normalized to their maximum programming stress.

Figure 9 shows typical results for the programming and recovery of samples with different HGM volume fractions. Following the compression results in the rubbery state, the programming stress depended on the applied strain and HGM volume fraction. Since the recovery stress also relied on these two parameters, the plotted programming stress and strain are normalized for each test. The recovery stress for each sample is normalized to its maximum programming stress. Since the recovery efficiency was studied here, the measured load in constrained recovery is shown in percentage. Note that the stress and strain plotted here are all engineering values. It is also noteworthy that the recovery times for all samples were also very similar and were independent of the HGM volume fraction. It took almost 50 min for the maximum stress to be achieved before the stress relaxation became dominant. Since programming samples without causing damage would increase the recovery efficiency and since different HGM volume fractions affected the compression behavior at 60 °C, the maximum possible programming stress–strain depends on Φ.

A significant drop in the true stress vs true strain plots previously denoted the failure in samples. In the engineering stress vs strain plots, the maximum programming strain can also be detected as the point where the rate of change in stress reduces to zero. Therefore, active monitoring the slope can be used to find the limit. However, as Figure S36 depicts, there might be no peak in the engineering stress vs strain plots for Φ < 50%. The failure point for these samples had to be determined experimentally. The performed tests indicated that strains up to 30% for the pure SMV and 25% for Φ = 40% did not reduce the recovery stress efficiency. However, surpassing the maximum stress limit decreased efficiency from the 80% found in Figure 9. For example, the effect of loading foam after the limit is depicted in Figures S37 and S39 for 50% syntactic foam. In Figure S37, programming is stopped before the peak. As expected, stress is recovered at a high 85% efficiency. However, when programming continued past the peak, as shown in Figure S38, the efficiency was decreased to 68%. If the sample was not crushed or broken, the extent to which the loading continued past this threshold did not affect the recovery efficiency, as shown in Figure S39. However, comparing the results shows a much faster decline in stress recovery for the more extreme programming. This decline is believed to be due to the propagation of the created small cracks in the last test, which accelerated the stress relaxation previously dominated by the polymer’s viscoelastic behavior.

All samples, programmed before reaching their stress peak, sprung back 5.5–7.5% relative to their initial length after unloading. It is understood that at identical strains, the stiffer and more elastic syntactic foams with higher ratios of elastic HGM particles relative to viscoelastic polymer must have a greater considerable springback. The results also showed an increase in the springback of samples with higher volume fractions relative to their programming strain. However, as long as the prepared foams were programmed close to their maximum stress and programming stopped before the load peaks, they all springback by an average strain of ∼6% upon unloading. The similarity in the springback strain highlights the relationship between the stiffness, elasticity, and yield stress–strain of the samples, which determines the stress peak. Usually, the springback decreased considerably when programming continued slightly after the peak (e.g., as illustrated in Figure S38). This is believed to be due to the possibility of strain energy release through the created cracks inside the sample. Interestingly, if the programming continued until the stress–strain curve slope became positive again (e.g., Figure S39), the springback increased again and usually exceeded the average springback strain observed when the programming was stopped before the peak. Figure S40 shows a specimen at different stages of the programming and recovery process.

### 3.10 Self-Healing Properties

The DCN-PEI polymer used as the syntactic foam’s matrix is designed to contain ester groups with dynamic exchange capability.^56^ Using the nitrogen atom as an internal catalyst, the β-hydroxy ester groups shown in Figure S41 can undergo transesterification and create new ester groups at a 150 °C curing temperature. Activating this dynamic ester exchange can be exploited to heal the SMV at elevated temperatures. Additionally, numerous hydrogen bonds are formed in the network due to the presence of unexhausted primary and secondary amine terminals, the ester groups, and the created hydroxyls. The recovery of these noncovalent hydrogen bonds after damage leads to the healing of the polymer at lower temperatures. The glass transition region was shown to be shifted by changing the ratio of the two monomers. The presented results showed that the room temperature was within the glass transition region for the current 1 to 1 ratio polymer and syntactic foam. Therefore, both the pure SMV and the syntactic foam at room temperature exhibited relatively high chain mobility. The accelerated reconfiguration of the network assists in reconnecting the cleaved hydrogen bonds. The catalytic action of the tertiary amines also enables the polymer to be dissolved in alcohols, which assists in its recyclability.

**Figure 10:**
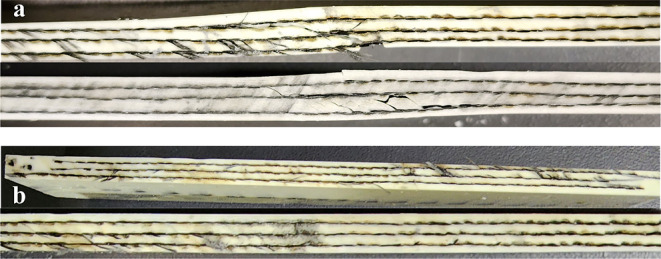
(a) Damaged laminates after impact and (b) damaged laminates after healing at room temperature for 72 h.

After the low-velocity impact, the syntactic foam matrix fractures because of the generated longitudinal and transverse shear stress in the beam during impact. However, due to the strong bonding between the syntactic foam and the plain-woven fabric, no delamination was seen. Instead, out-of-plane shear cracks were seen. A set of laminates after the impact test can be seen in Figure 10a.

To evaluate the healing efficiency, all samples underwent an impact test. The laminates were healed, and their second impact test results were compared with those of the unhealed control group. Based on the maximum impact force (*F*_max_) during impact, the calculated energy was divided into two parts: the portion with increasing load was attributed to crack initiation, and the portion with decreasing impact force was associated with crack propagation. The healing efficiency is calculated by the difference between the measured crack initiation energies (*E*_Fmax_) of the healed samples with those unhealed compared to the difference in this energy between damaged and undamaged laminate. The healing efficiency can be formulated as
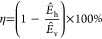
6where *Ê*_h_ and *Ê*_v_ are the normalized energy losses for the healed and virgin samples and are calculated as
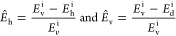
7where *E*^i^ is the crack initiation energy. Subscripts h–v denote healed, unhealed damaged, and undamaged virgin specimens, respectively. The normalized values are used to calculate the healing efficiency to offset the effect of slight differences in the geometries of samples on the measured impact energy. The conducted tests demonstrated that the healing efficiency at room temperature could reach up to 61.8%.

Figure 10b shows the laminates posthealing at room temperature for roughly 72 h under an approximate stress of 3 MPa. Compared with the damaged ones, these samples hardly show any sign of previous damage, even after careful inspection.

The impact test results show the difference between the damaged and undamaged specimens and healed and undamaged specimens in Figures S42 and S43, respectively. The healed laminate absorbed more energy than the damaged one due to the re-established hydrogen bonds in the fractured zone. Please note that the results are normalized before plotting, following the method used to calculate the healing efficiency. The measured force is normalized by the maximum force of the undamaged sample (*F*_max_), and the energy calculated by the instrument is normalized by the energy at the maximum force (*E*_Fmax_) for the virgin laminate. Here, *Ê*_h_ = 11.95% and *Ê*_v_ = 31.29%.

The healing tests were repeated at 60 °C, close to the syntactic foam’s glass transition zone. The healing results showed no significant change compared with the room-temperature efficiencies. However, the healing time and healing pressure of the tests could be reduced to 24 h and 1 MPa, respectively. The healing mechanism is believed to be due to the re-establishment of the hydrogen bonds. Therefore, the healing efficiency is similar to healing at room temperature but with a much shorter healing time and less healing pressure. This change is because, within the glass transition zone, the mobility of the SMV network is much higher than that at lower temperatures. Since the polymer chains had higher mobility at elevated temperatures, a lower pressure and a shorter time were enough to position the hydrogen bonds within the critical distance. Higher stress and longer time were required to accomplish this at room temperature.

Reactivating the transesterification reaction between the PEI cross-linker and the DCN monomer at high temperatures further increased the healing efficiency of the laminate. A damaged laminate was first healed at room temperature for 36 h. After room-temperature healing, it was pressed at 150 °C by nearly 1 MPa for 2 h. The results shown in Figure S44 compare the measured impact force and energy for this laminate before and after healing. The reformed covalent bonds increased the calculated efficiency to 71.73%, about 10% higher than room-temperature healing.

## 4 Conclusions

This study introduced a new lightweight shape memory vitrimer-based syntactic foam with unique intrinsic self-healing properties. By exploiting a polymer matrix with two types of reversible bonds in its network, the presented foam offers dual self-healing mechanisms that act at both room and elevated temperatures. Despite its extremely low density, the test results demonstrated remarkable thermal and mechanical properties of this syntactic foam.

This article also comprehensively discussed the impact of increasing the volume fraction of HGMs beyond the typical 40% on various properties of shape memory vitrimer-based syntactic foams. Theoretically and practically, fabricating syntactic foams with volume fractions exceeding 70% of glass bubbles appears virtually impossible. However, although higher volume fractions result in lighter materials, the shape memory effect notably declines at the 60% volume fraction. The mechanical properties of the syntactic foam were also proven to reduce substantially at the 70% volume fraction due to the minimal polymer matrix around the microspheres. These systematic experimental studies aid scientists and engineers in better understanding the effects of HGM volume fraction on the thermomechanical and functional properties of syntactic foams, guiding them in designing better and smarter materials.

## Supporting Information Available

Morphological, spectroscopic, and thermal data; rheological and mechanical properties; viscoelastic behavior and analysis; programming and recovery behavior; volume fraction effects on HGMs; SMV structure and stability (PDF)
