# Supplementary material for: On Lightweight Shape Memory Vitrimer Composites
Source: ACS Appl Polym Mater. 2023 Dec 15;6(1):154–69. doi: 10.1021/acsapm.3c01749 (PMC10788861; doi:10.1021/acsapm.3c01749)
Supplement: Supplementary file 1 — ap3c01749_si_001.pdf [file ap3c01749_si_001.pdf]

Supporting Information

On lightweight shape memory vitrimer composites

Siavash Sarrafan, Guoqiang Li[*]

Department of Mechanical & Industrial Engineering, Louisiana State University, Baton Rouge, LA 70803, USA

***Corresponding Author: lguoqi1@lsu.edu**

Table S1. The porosity of the prepared samples with their calculated theoretical densities (ρ_{th}), the actual measured densities (ρ_a), the difference between the two densities, and the division of this difference by the volume fraction of the SMV in the material ($V_{SMV}/V_T = (\boldsymbol{\rho_a} - \boldsymbol{\rho_{th}})/\phi_{SMV}$) and mass of the SMV in the material (m_{SMV}/m_T).

$\boldsymbol{\phi_{HGM}}$ (%)	Porosity (%)	$\boldsymbol{\rho_{th}}$ (g/cc)	$\boldsymbol{\rho_a}$ (g/cc)	$\boldsymbol{\rho_a - \rho_{th}}$ (g/cc)	$\frac{\boldsymbol{\rho_a - \rho_{th}}}{\phi_{SMV}}$ (g/cc)	$\frac{\boldsymbol{\rho_a - \rho_{th}}}{w_{SMV}}$ (g/cc)
0	0	1.129	1.198	0.0694	0.0694	0.06937
40	37.6	0.737	0.801	0.0638	0.1064	0.06947
50	47	0.639	0.697	0.0577	0.1154	0.06535
60	56.4	0.541	0.593	0.0519	0.1299	0.06230
70	65.8	0.444	0.490	0.0461	0.1538	0.06044

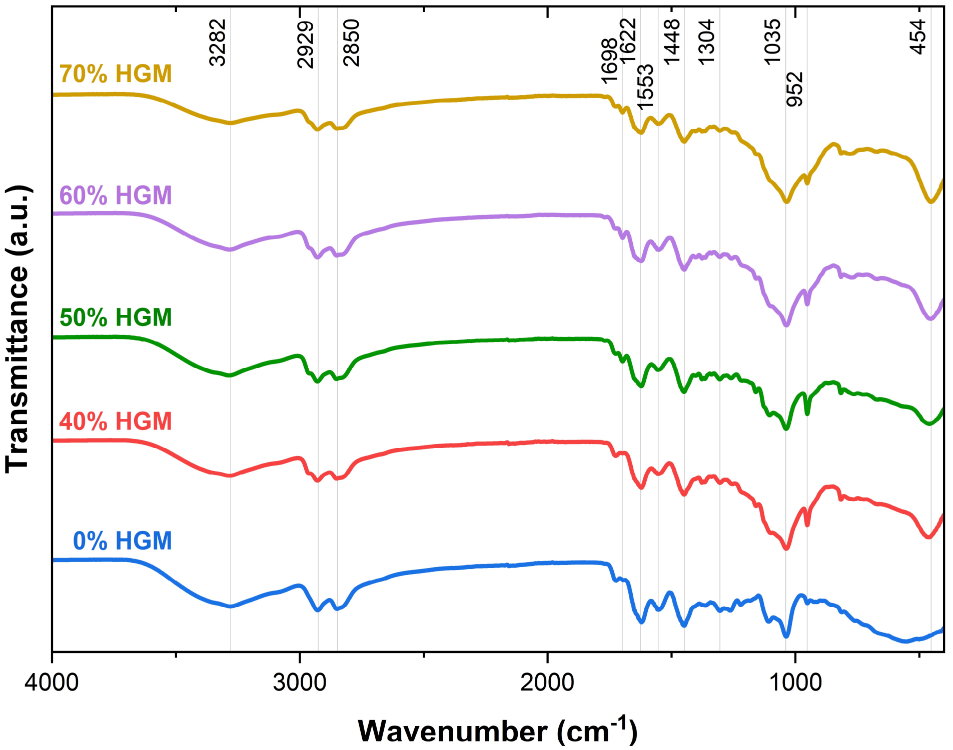

Figure S1. Comparison of the FTIR spectrum for all five prepared samples. The observed peaks are almost identical among them.

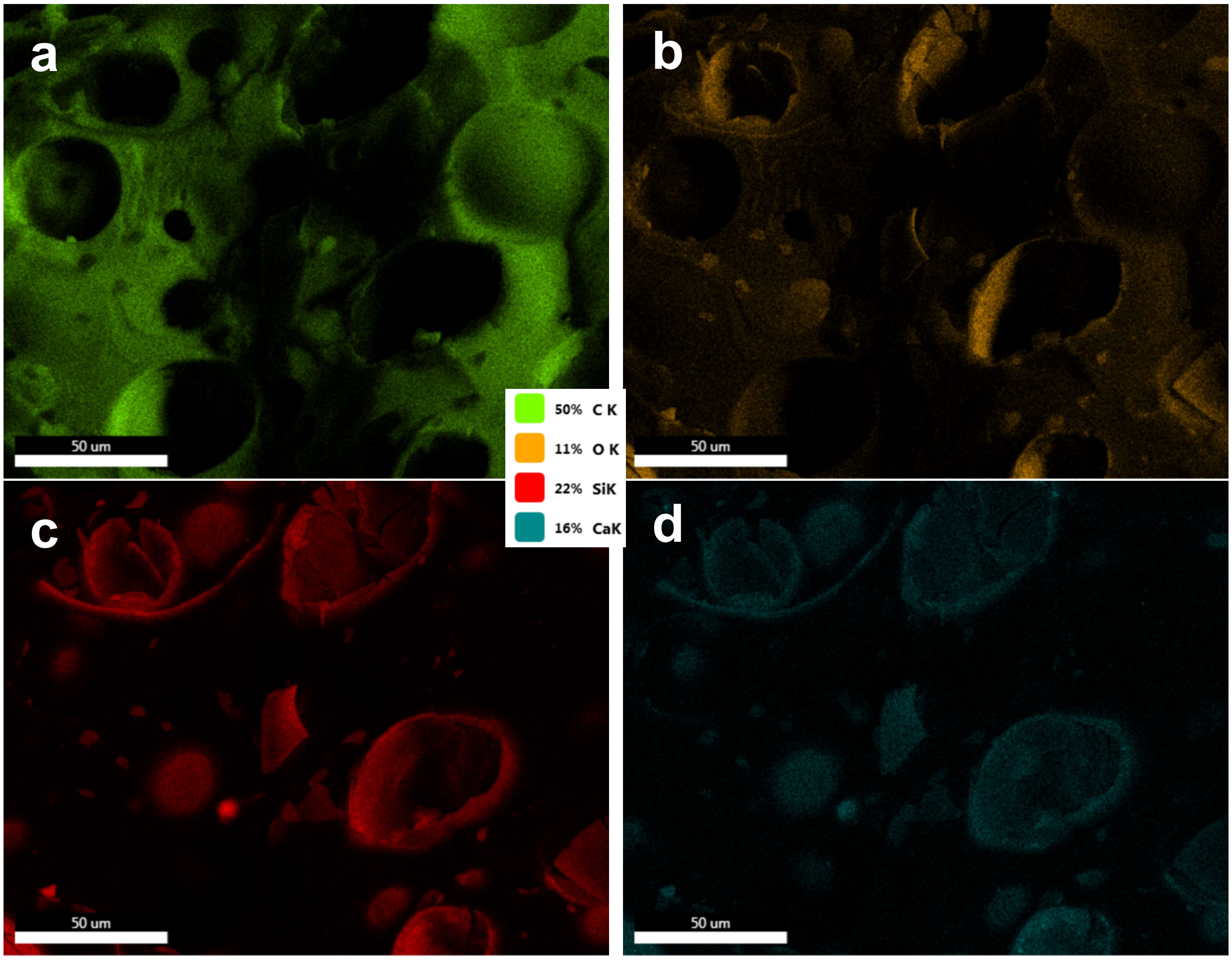

Figure S2. Individual element maps for a) carbon, b) oxygen, c) silicon, and d) calcium elements on the surface of the syntactic foam using EDS.

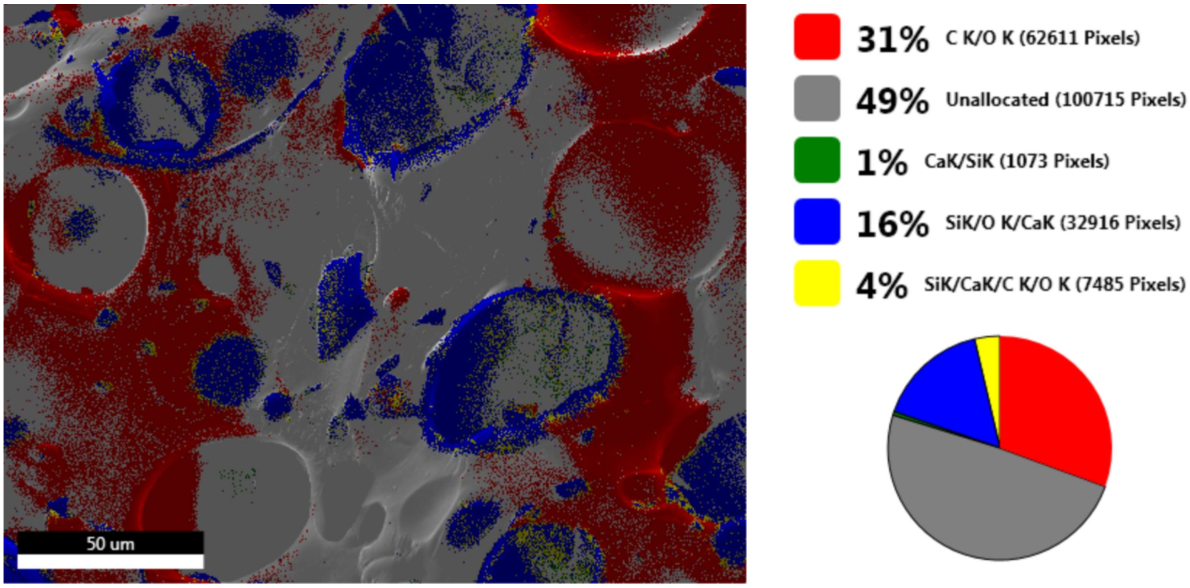

Figure S3. EDS phase and element area map for the syntactic foam, showing their ratio on the analyzed area.

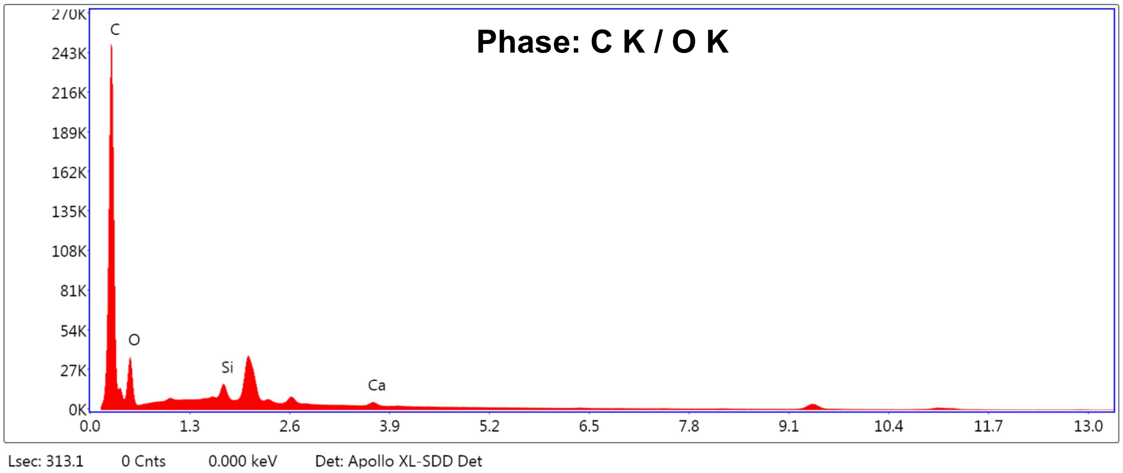

Figure S4. EDS analysis spectrum of carbon/oxygen phase on the surface of the syntactic foam.

Table S2. ZAF-corrected quantitative results of the EDS analysis for the carbon/oxygen phase on the surface of the syntactic foam.

Element	*Weight (%)*	*Atomic (%)*	*Net Intensity*	*Error (%)*	*K ratio*	*Z*	*R*	*A*	*F*
C K	73.85	79.45	6408.30	4.22	0.5058	1.0126	0.9940	0.6764	1.0000
O K	24.68	19.93	1009.10	9.79	0.0315	0.9673	1.0123	0.1319	1.0000
SiK	1.08	0.50	493.90	2.54	0.0084	0.8767	1.0520	0.8726	1.0073
CaK	0.38	0.12	106.50	3.89	0.0035	0.8264	1.0780	1.0405	1.0587

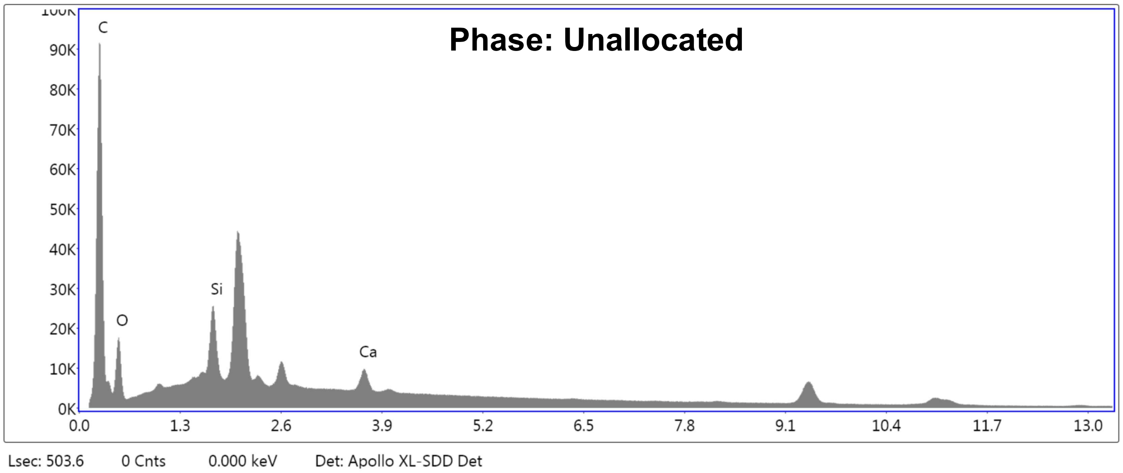

Figure S5. EDS analysis spectrum of the unallocated phase on the surface of the syntactic foam.

Table S3. ZAF-corrected quantitative results of the EDS analysis for the unallocated phase on the surface of the syntactic foam.

Element	*Weight (%)*	*Atomic (%)*	*Net Intensity*	*Error (%)*	*K ratio*	*Z*	*R*	*A*	*F*
C K	71.35	78.15	1486.80	5.08	0.4313	1.0162	0.9915	0.5948	1.0000
O K	24.49	20.13	271.50	9.97	0.0311	0.9709	1.0101	0.1309	1.0000
SiK	2.51	1.18	311.80	2.63	0.0194	0.8803	1.0502	0.8695	1.0075
CaK	1.65	0.54	123.90	3.22	0.0148	0.8299	1.0767	1.0358	1.0441

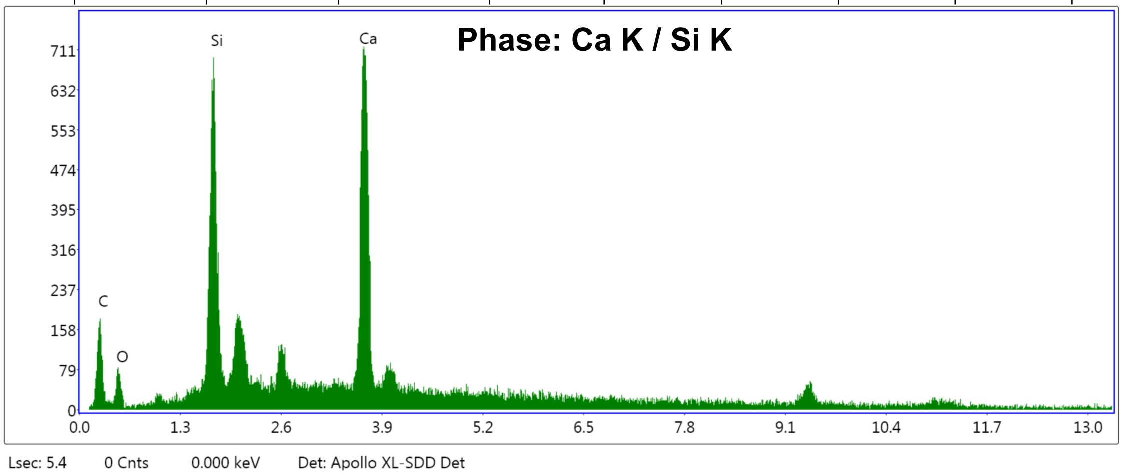

Figure S6. EDS analysis spectrum of calcium/silicon phase on the surface of the syntactic foam.

Table S4. ZAF-corrected quantitative results of the EDS analysis for the silicon/calcium phase on the surface of the syntactic foam.

Element	*Weight (%)*	*Atomic (%)*	*Net Intensity*	*Error (%)*	*K ratio*	*Z*	*R*	*A*	*F*
C K	35.04	54.04	220.70	12.10	0.1076	1.0818	0.9476	0.2839	1.0000
O K	18.97	21.97	104.40	16.02	0.0201	1.0368	0.9694	0.1023	1.0000
SiK	13.83	9.12	1017.20	5.66	0.1063	0.9448	1.0175	0.8021	1.0137
CaK	32.16	14.86	1433.80	3.40	0.2878	0.8930	1.0508	0.9906	1.0117

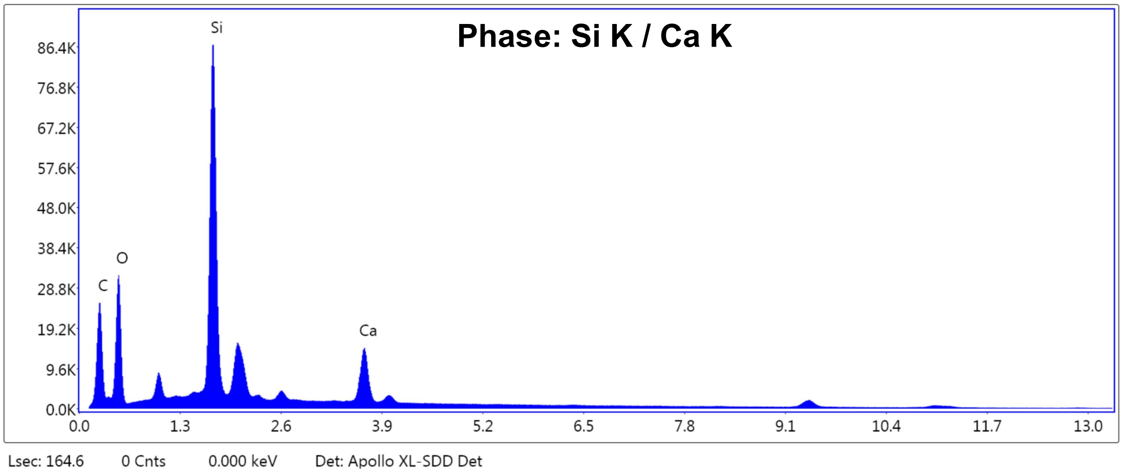

Figure S7. EDS analysis spectrum of silicon/calcium phase on the surface of the syntactic foam.

Table S5. ZAF-corrected quantitative results of the EDS analysis for the silicon/calcium phase on the surface of the syntactic foam.

Element	*Weight (%)*	*Atomic (%)*	*Net Intensity*	*Error (%)*	*K ratio*	*Z*	*R*	*A*	*F*
C K	43.33	54.81	1199.50	8.43	0.1243	1.0426	0.9765	0.2752	1.0000
O K	37.45	35.56	1523.50	9.45	0.0624	0.9972	0.9964	0.1672	1.0000
SiK	14.50	7.84	4944.90	2.79	0.1098	0.9056	1.0395	0.8321	1.0055
CaK	4.72	1.79	965.00	1.65	0.0412	0.8544	1.0684	0.9968	1.0247

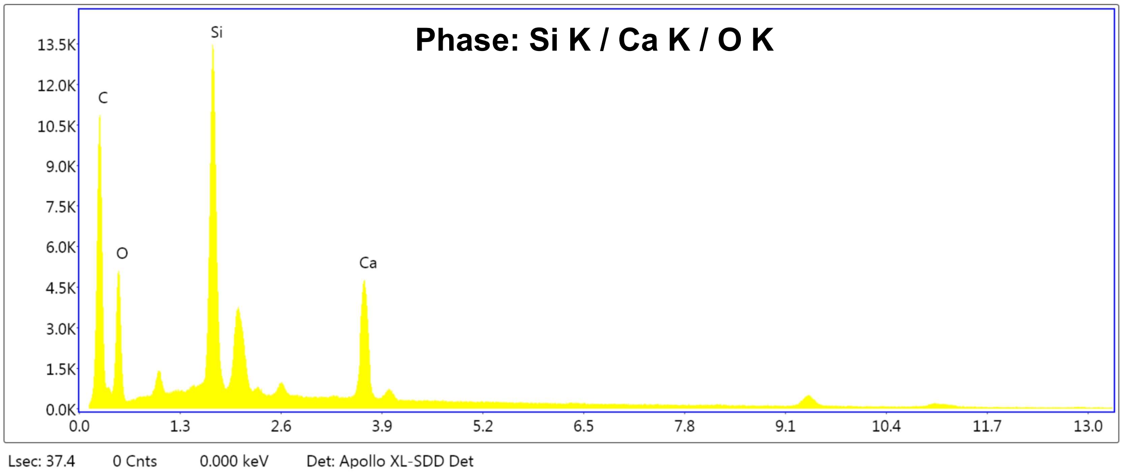

Figure S8. EDS analysis spectrum of silicon/calcium/oxygen phase on the surface of the syntactic foam.

Table S6. ZAF-corrected quantitative results of the EDS analysis for the silicon/calcium/oxygen phase on the surface of the syntactic foam.

Element	*Weight (%)*	*Atomic (%)*	*Net Intensity*	*Error (%)*	*K ratio*	*Z*	*R*	*A*	*F*
C K	55.56	66.70	2232.30	7.61	0.2124	1.0337	0.9806	0.3697	1.0000
O K	29.46	26.55	1082.00	10.11	0.0407	0.9884	1.0001	0.1397	1.0000
SiK	8.87	4.55	3325.10	2.87	0.0678	0.8973	1.0424	0.8454	1.0074
CaK	6.10	2.20	1371.00	2.13	0.0537	0.8465	1.0707	1.0139	1.0249

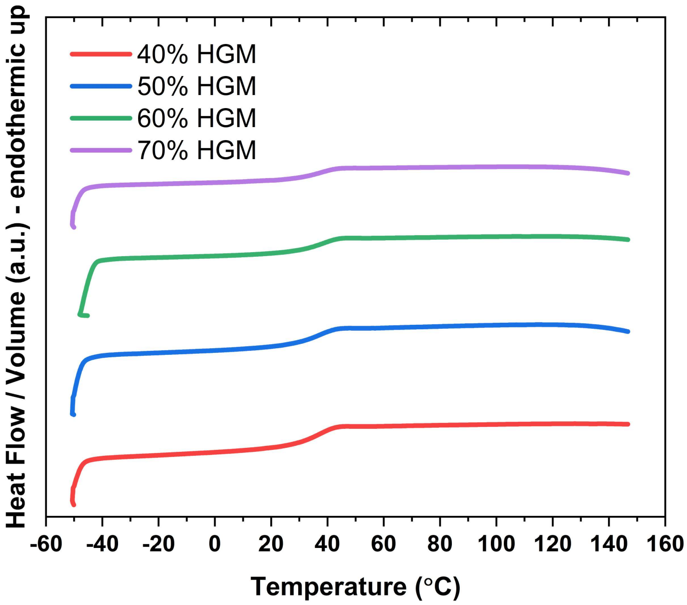

Figure S9. DSC thermogram of syntactic foams prepared with different volume fractions of glass microspheres comparing the glass transition temperature during the second heating segment.

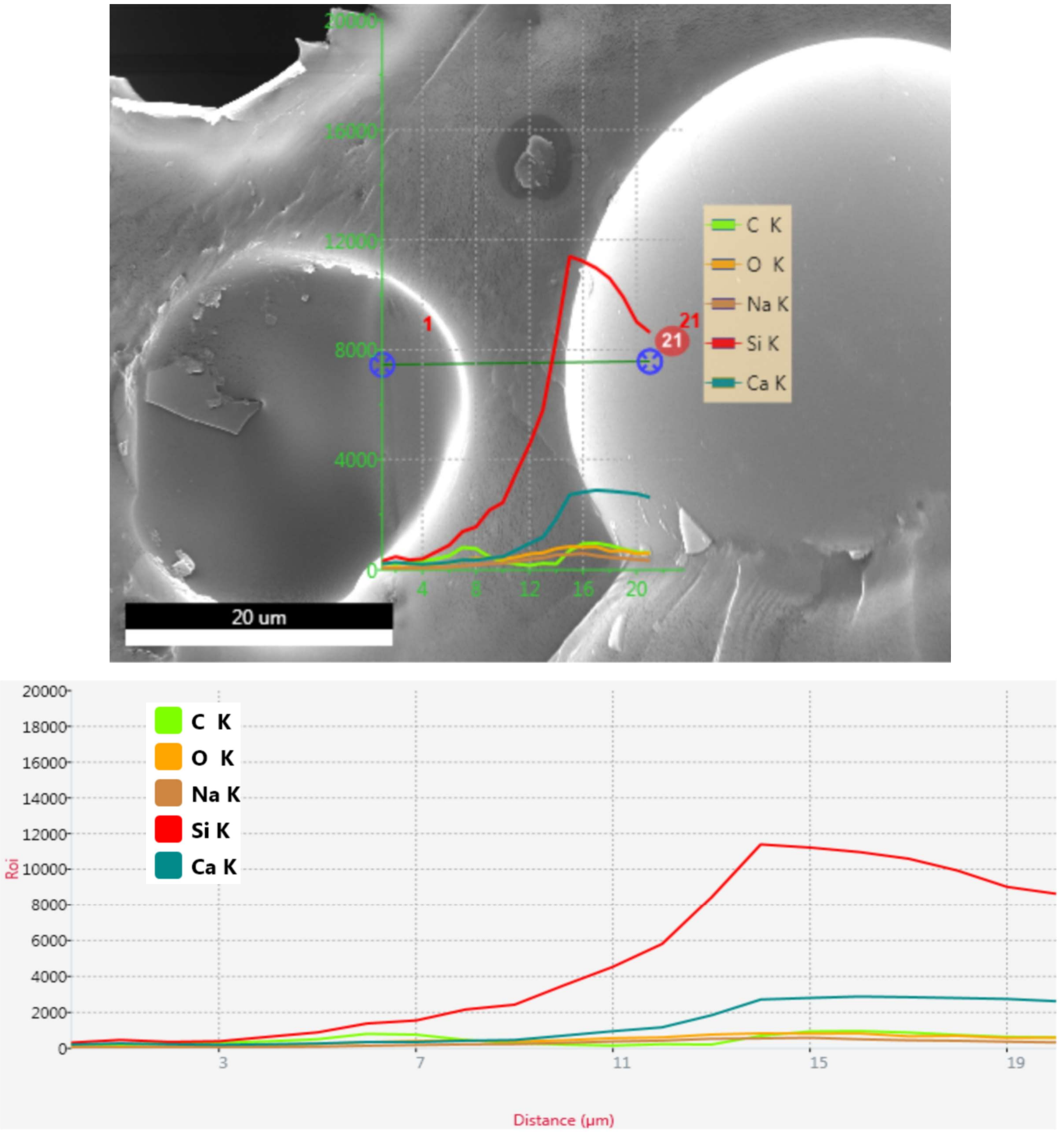

Figure S10. An SEM image with a path used by EDS to analyze the change in elements in the area near the glass bubbles on the surface of prepared syntactic foams and the energy intensity of each element measured at different points across a hollow glass bubble in a syntactic foam.

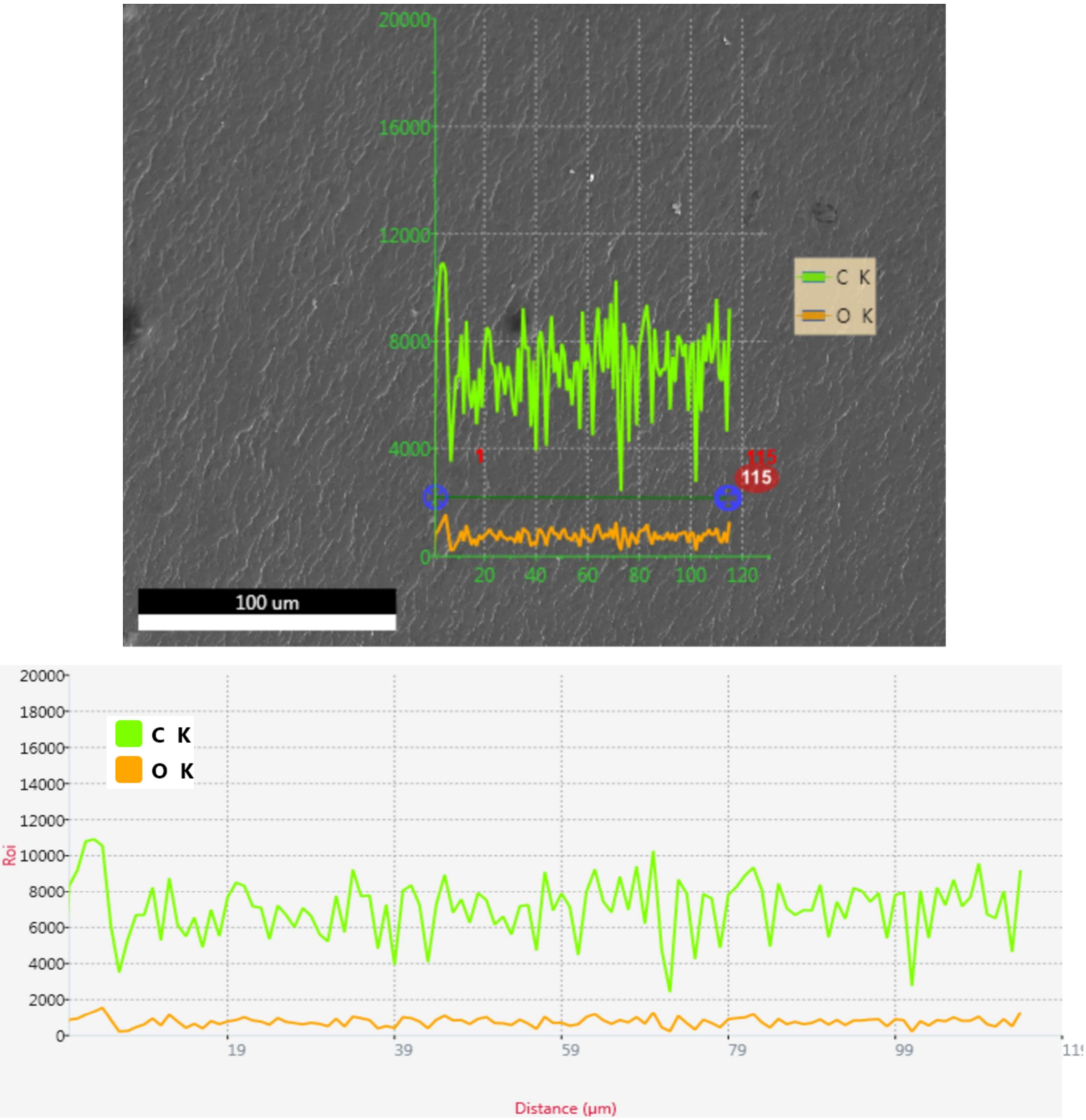

Figure S11. An SEM image with a path used by EDS to analyze the change in elements in the SMV matrix and the energy intensity of each element measured on this path as the control.

Table S7. The storage modulus (E′) and the loss modulus (E″) of samples with different HGM volume fractions at three different temperatures extracted from the temperature sweep tests. These temperatures were chosen to represent the frozen state, room temperature, and rubbery state.

Volume Fraction	-20 °C		30 °C		100 °C	
Φ (%)	$\boldsymbol{E'}$ (GPa)	$\boldsymbol{E''}$ (GPa)	$\boldsymbol{E'}$ (GPa)	$\boldsymbol{E''}$ (MPa)	$\boldsymbol{E'}$ (MPa)	$\boldsymbol{E''}$ (MPa)
0	7.92	2.36	2.12	546.19	4.71	0.33
40	4.66	0.28	1.74	296.10	17.54	0.62
50	3.79	0.32	1.15	238.74	20.18	0.94
60	2.39	0.287	1.01	231.86	22.18	2.17
70	1.57	0.08	0.95	141.11	24.56	5.38

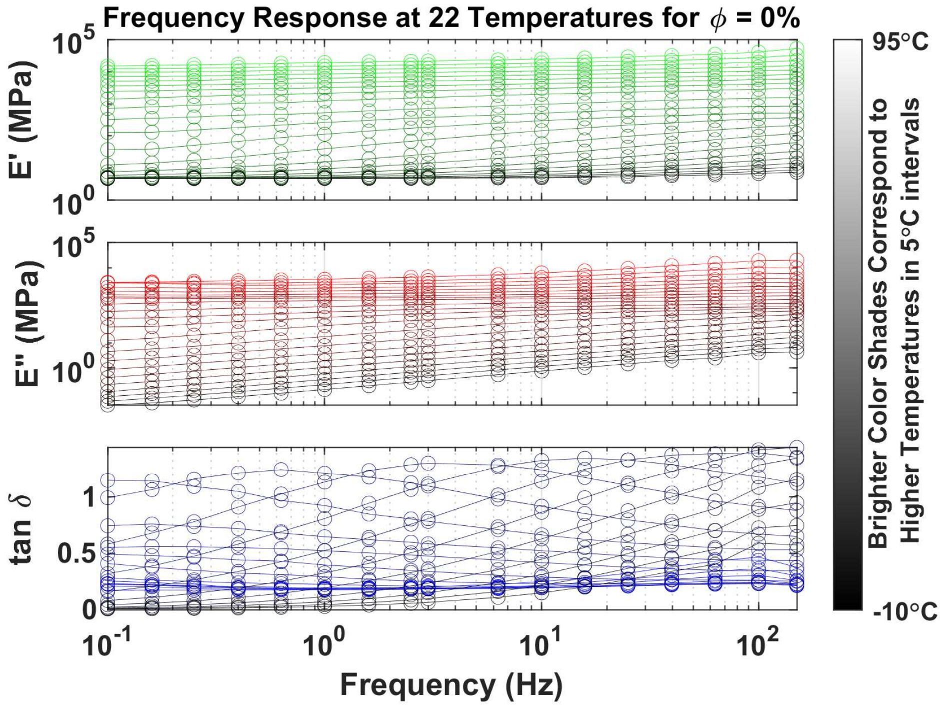

Figure S12. The rheological properties (E', E'', $\tan\delta$) of the pure SMV at different frequencies and different temperatures.

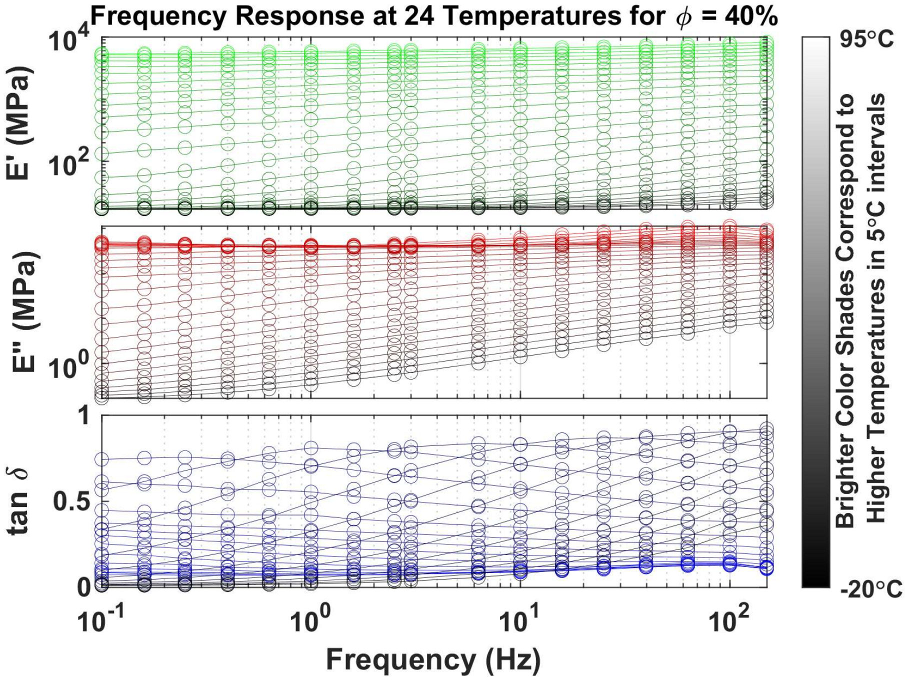

Figure S13. The rheological properties (E', E'', $\tan\delta$) of the syntactic foam with 40% HGMs by volume at different frequencies and different temperatures.

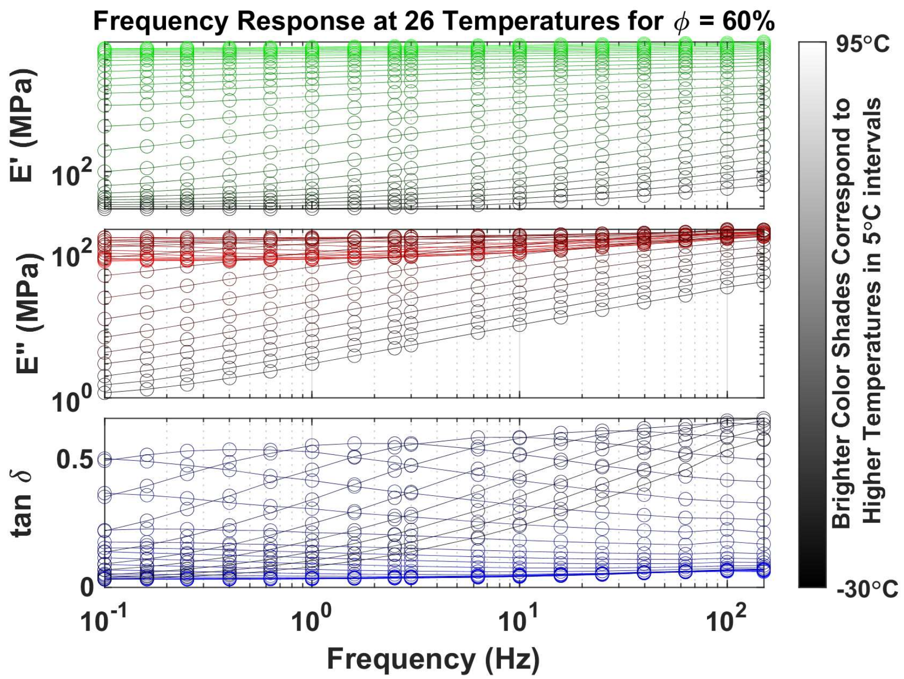

Figure S14. The rheological properties (E', E'', $\tan\delta$) of the syntactic foam with 60% HGMs by volume at different frequencies and different temperatures.

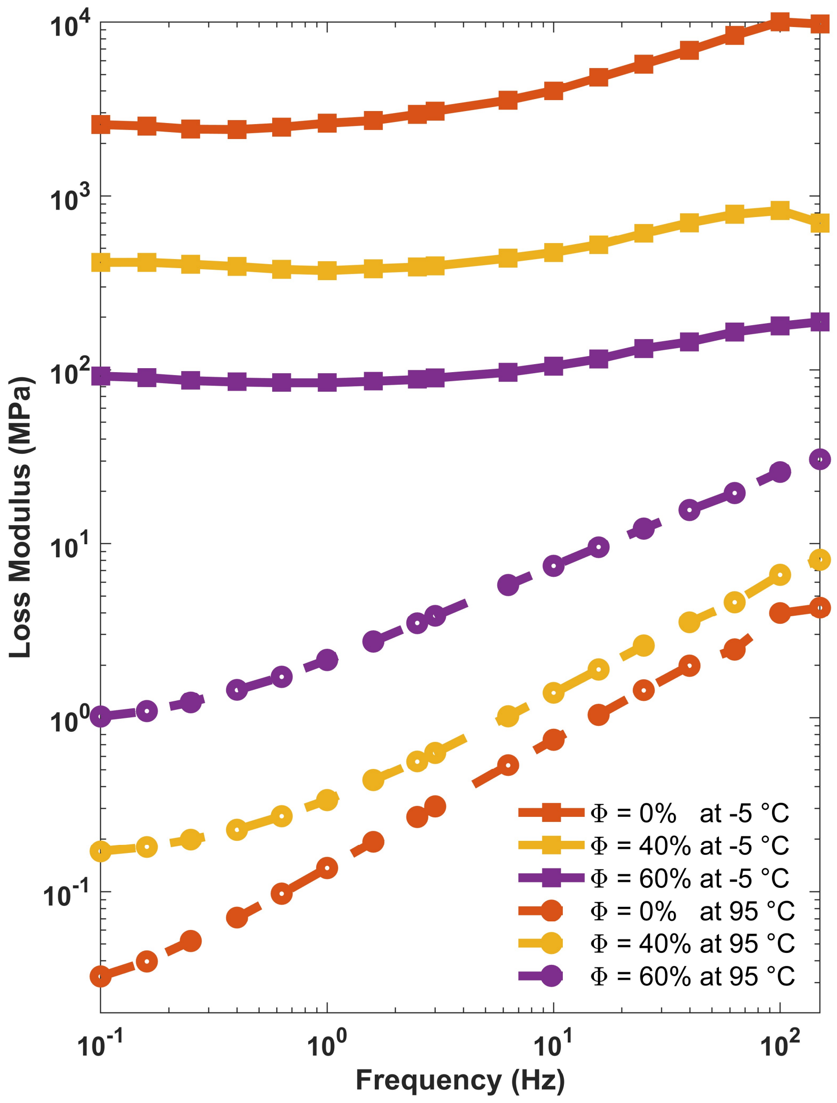

Figure S15. The effect of frequency on the loss modulus of the specimens with different levels of HGM volume fractions at the glassy state (-5 °C), shown by square markers, and at the rubbery state (95 °C), presented by circle markers.

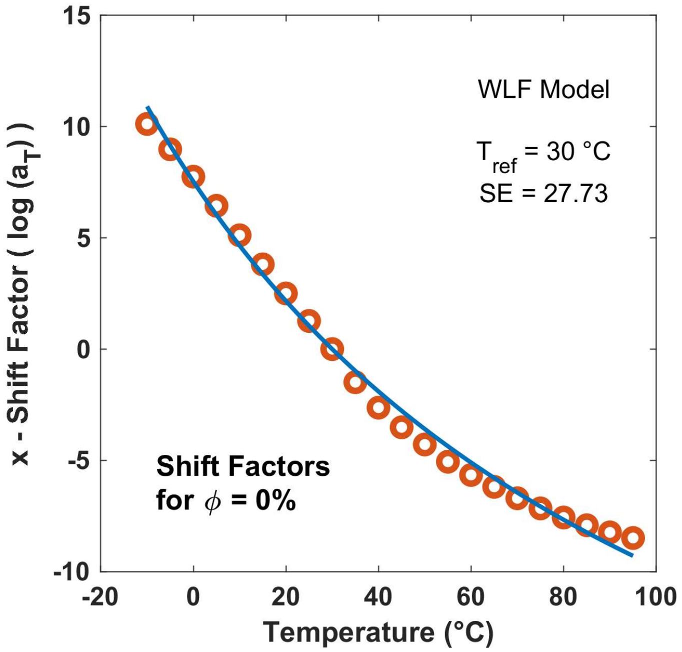

Figure S16. The shift factors for the time-temperature superposition analysis on the rheological properties at different frequencies for pure SMV at 30 °C. The shift factors are the same for the storage modulus, loss modulus, and loss factor. A WLF model is fitted to the shift factors and the calculated standard error is noted.

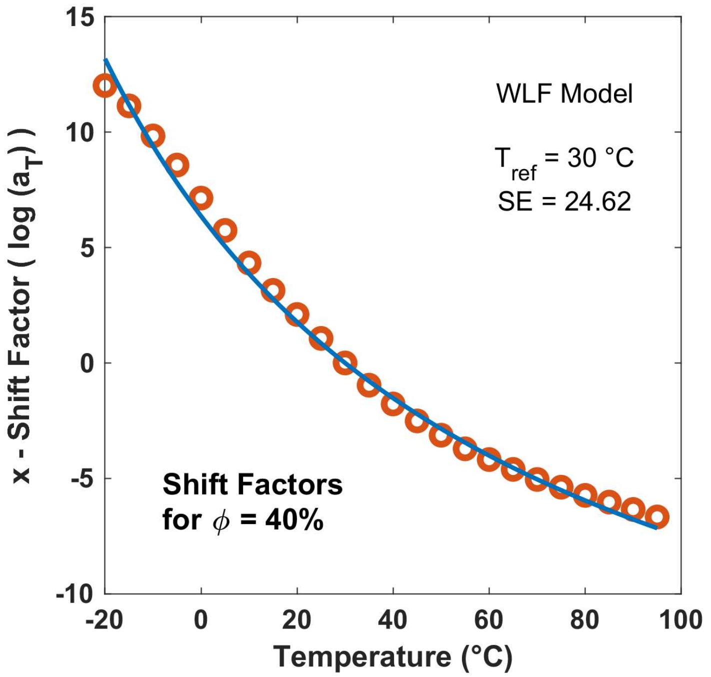

Figure S17. The shift factors for the time-temperature superposition analysis on the rheological properties at different frequencies for the syntactic foam with 40% HGMs by volume at 30 °C. The shift factors are the same for the storage modulus, loss modulus, and loss factor. A WLF model is fitted to the shift factors, and the calculated standard error is noted.

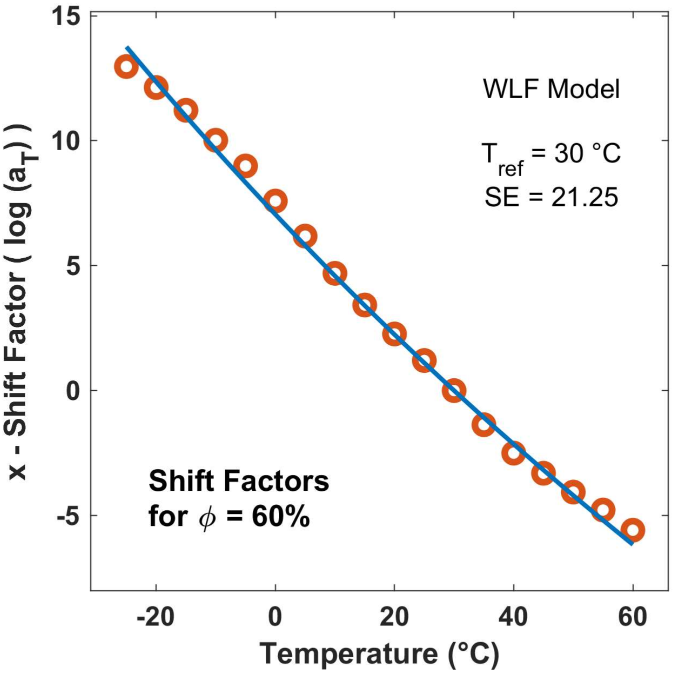

Figure S18. The shift factors for the time-temperature superposition analysis on the rheological properties at different frequencies for the syntactic foam with 60% HGMs by volume at 30 °C. The shift factors are the same for the storage modulus, loss modulus, and loss factor. A WLF model is fitted to the shift factors, and the calculated standard error is noted.

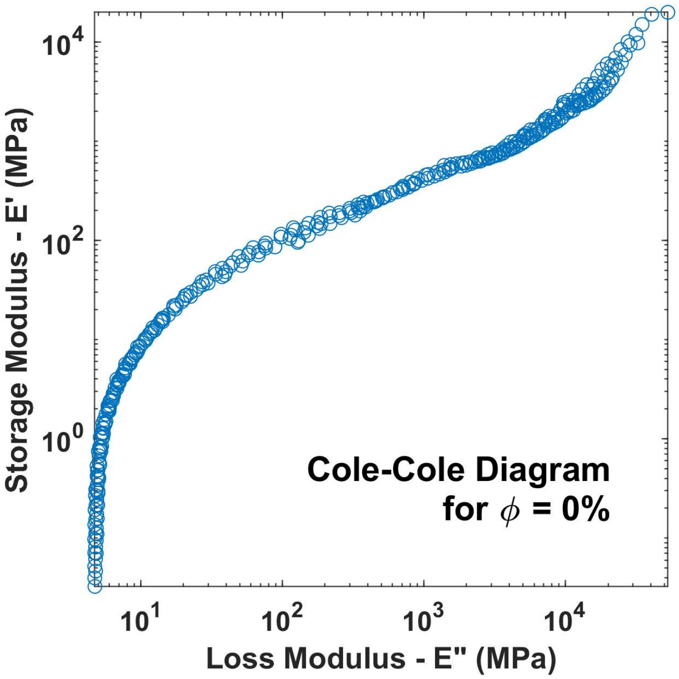

Figure S19. The smooth Cole-Cole diagram for the pure SMV suggests that it is rheologically simple and, therefore, TTS can be applied to it.

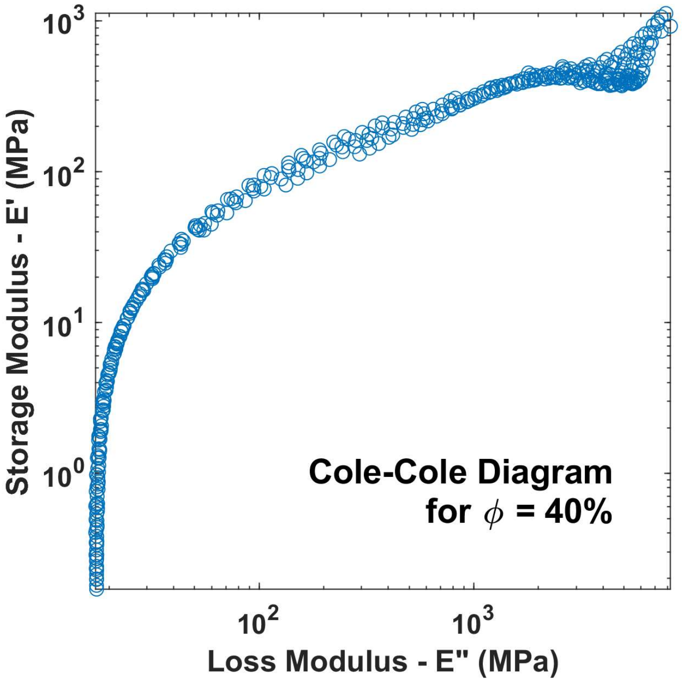

Figure S20. The smooth Cole-Cole diagram for the syntactic foam with 40% HGMs by volume suggests that it is rheologically simple and, therefore, TTS can be applied to it.

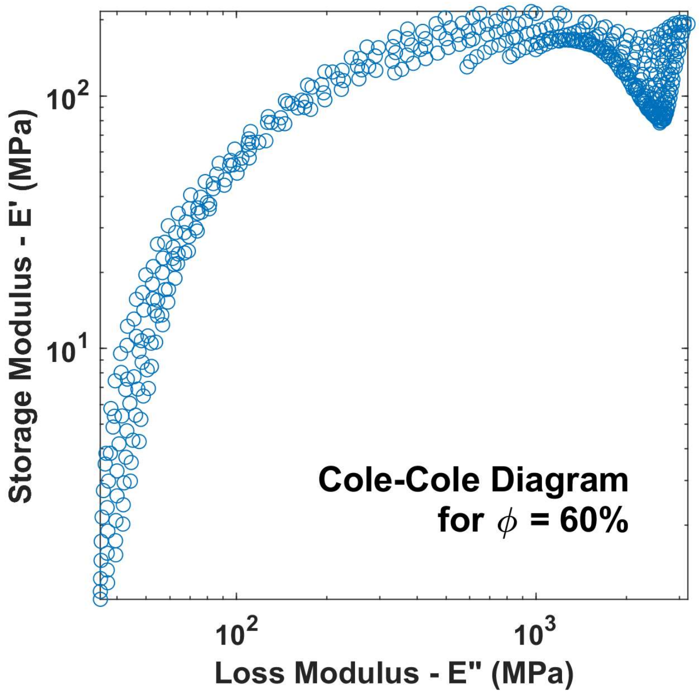

Figure S21. The smooth Cole-Cole diagram for the syntactic foam with 60% HGMs by volume suggests that it is rheologically simple and, therefore, TTS can be applied.

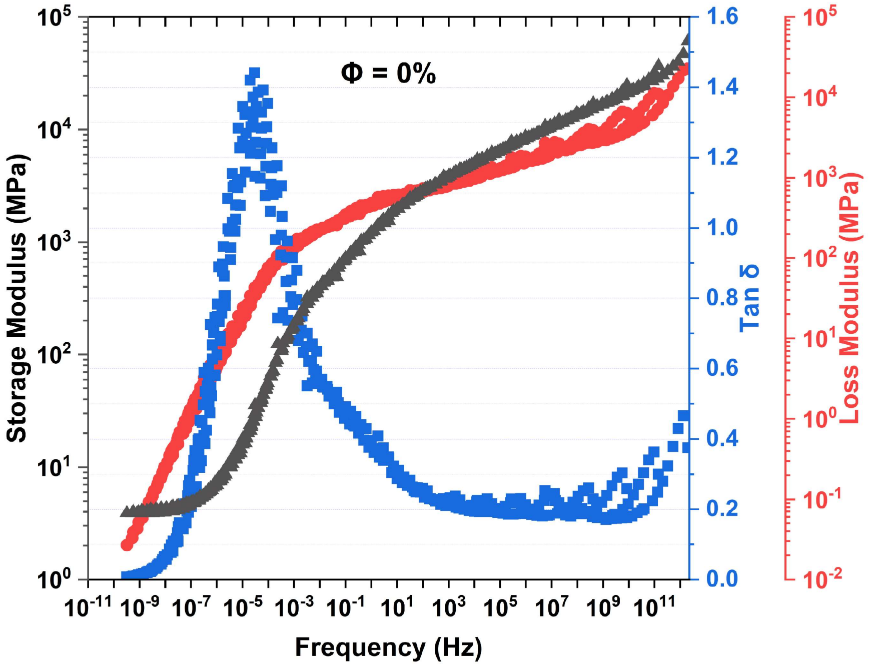

Figure S22. The master curve generated from the TTS analysis of the pure SMV's frequency response at 30 °C.

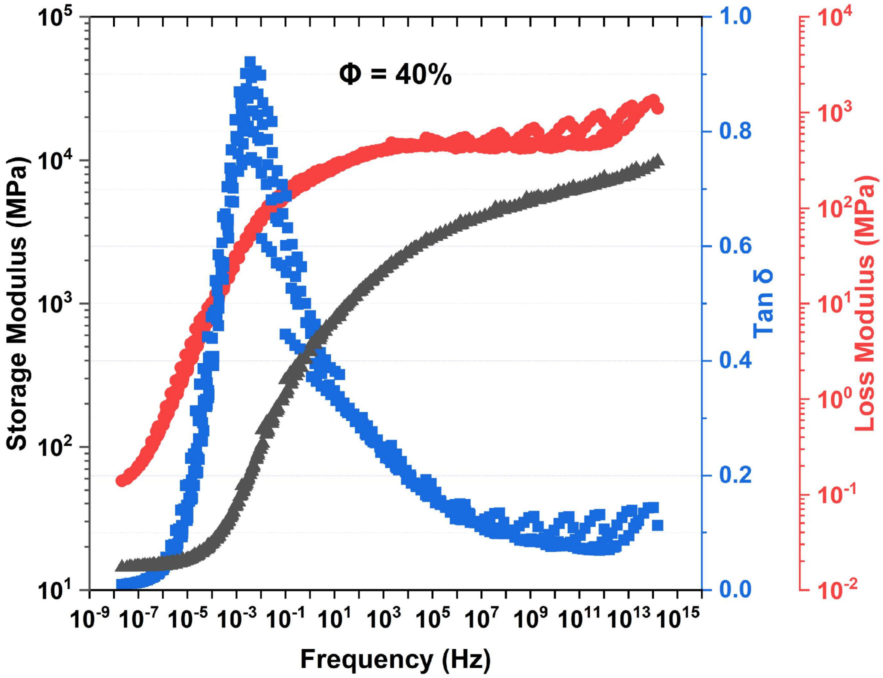

Figure S23. The master curve generated from the TTS analysis of the frequency response of the syntactic foam with 40% HGMs by volume, at 30 °C.

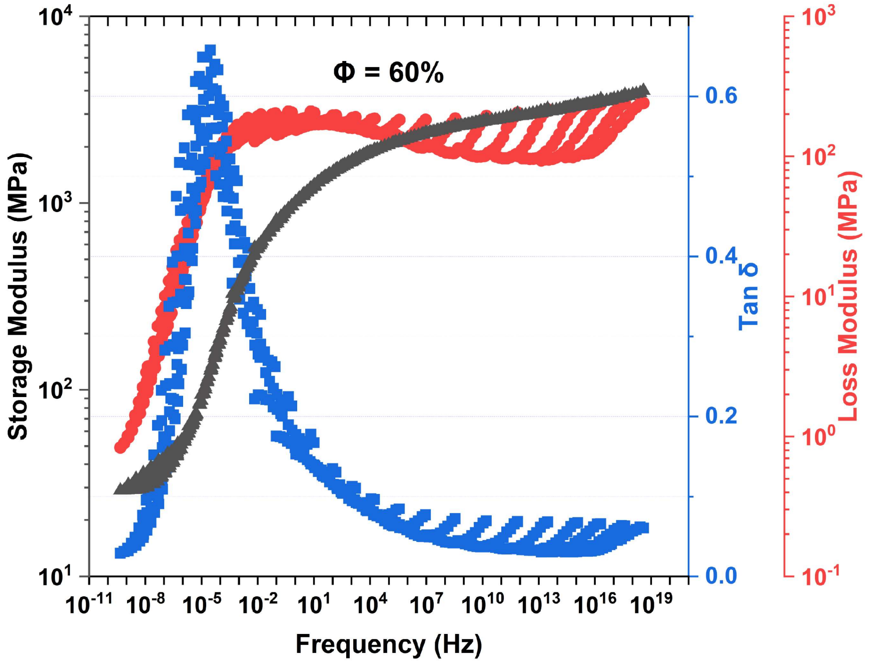

Figure S24. The master curve generated from the TTS analysis of the frequency response of the syntactic foam with 60% HGMs by volume, at 30 °C.

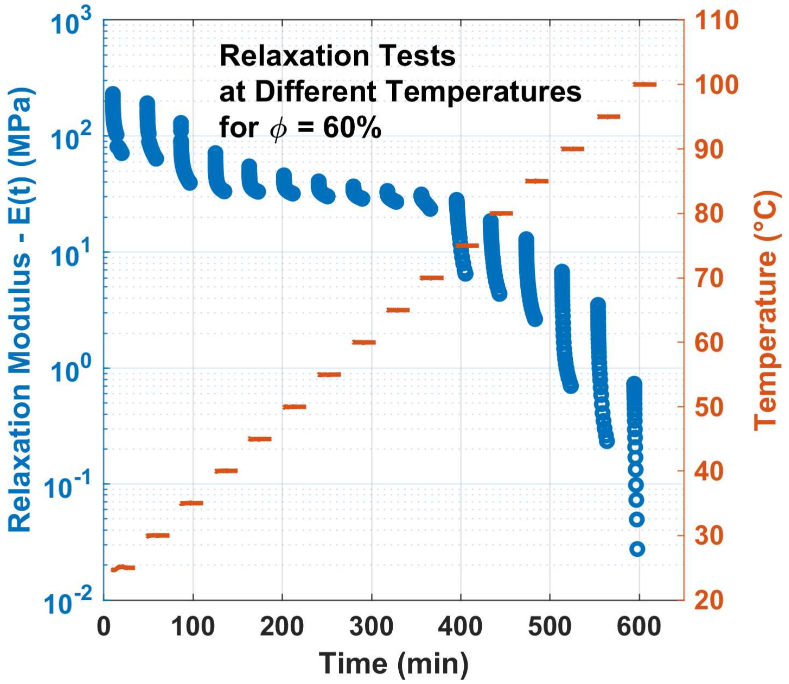

Figure S25. The relaxation tests performed at different temperatures at 5 °C for the TTS analysis. At each temperature, specimens were stretched for 10 minutes, and the required stress to stay elongated was measured over time. They were then unloaded for 15 minutes to recover before the next cycle. The figure above shows the results for the syntactic foam with 60% HGMs per volume.

Table S8. The discrete relaxation model spectrums for the pure polymer and the syntactic foams with Φ = 40 and 60% at a temperature close to their glass transition temperatures. The final row contains the relaxation modulus calculated through the TTS for its maximum time.

Φ (%)	*0*	*0*	*40*	*60*
T (°C)	60	65	60	60
E_1 *(MPa)*	5.14	5.14	13.2	32.5
$\boldsymbol{\tau_1}$ *(s)*	1.49E+10	6.95E+09	2.20E+11	6.92E+05
E_2 *(MPa)*	49.6	39.7	35.2	30.1
$\boldsymbol{\tau_2}$ *(s)*	0.06207	0.03521	0.02753	1.84
E_3 *(MPa)*	152	147	50.5	87
$\boldsymbol{\tau_3}$ *(s)*	5.40E-03	2.85E-03	5.73E-03	0.1285
E_4 *(MPa)*	284	266	74.3	71.4
$\boldsymbol{\tau_4}$ *(s)*	1.78E-04	9.7E-05	3.91E-03	3.87E-03
E_5 *(MPa)*	3.82E-07	3.45E-07	6.60E-06	6.75E-07
$\boldsymbol{\tau_5}$ *(s)*	0.2685	1.219	5.42	7.45E-04
E_6 *(MPa)*	1.24E-07	4.35E-08	8.96E-06	4.31E-09
$\boldsymbol{\tau_6}$ *(s)*	1.668	0.08259	9.354	8.57E-04
$E_{t\rightarrow\infty}$ *(MPa)*	5.01	5.01	13.2	29.2

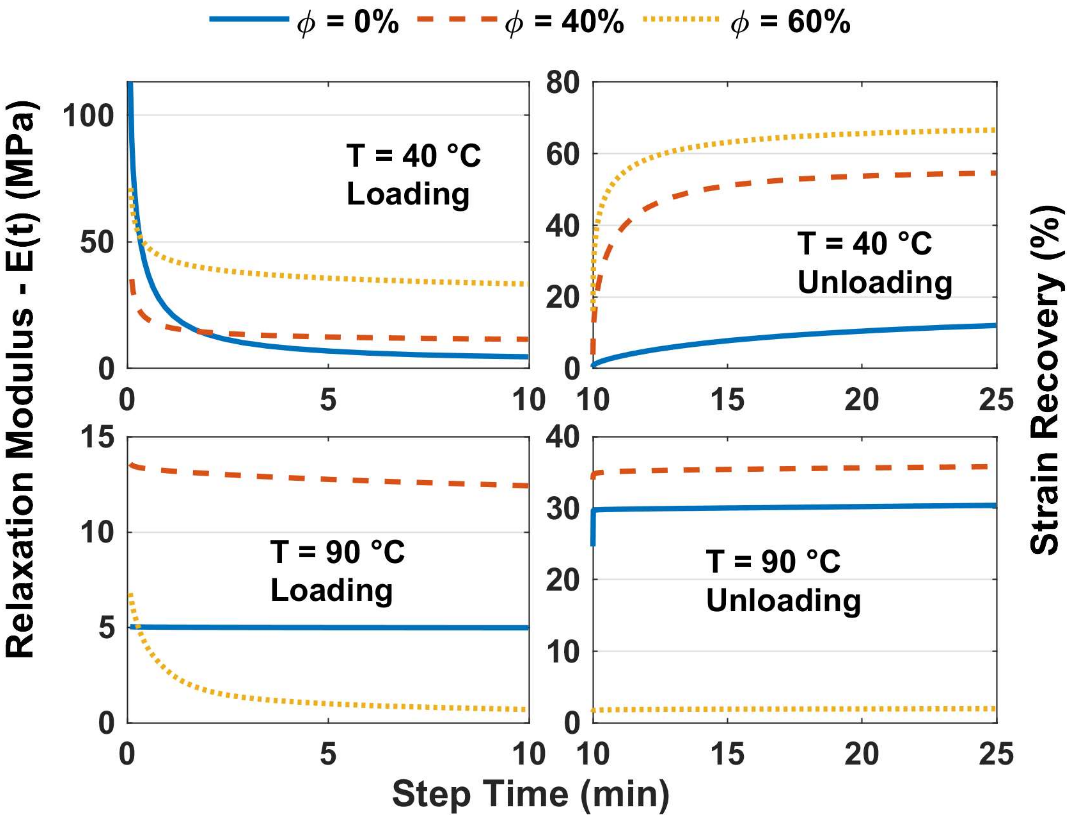

Figure S26. The effect of HGMs volume fraction on the viscoelastic behavior of the syntactic foams, compared to the pure vitrimer, at 40 and 90 °C. These temperatures were selected considerably below and above the glass transition temperature to represent the samples in their frozen and rubbery states. Specimens with Φ = 0, 40, and 60% were subjected to a constant strain during the first 10-min of each cycle and were then released to recover freely during the second 15 min. At each cycle, samples were soaked for 10 min at the corresponding temperature to reach equilibrium before this loading/unloading process. The two plots on the left show the relaxation modulus during loading, and the two on the right display the strain recovery during unloading.

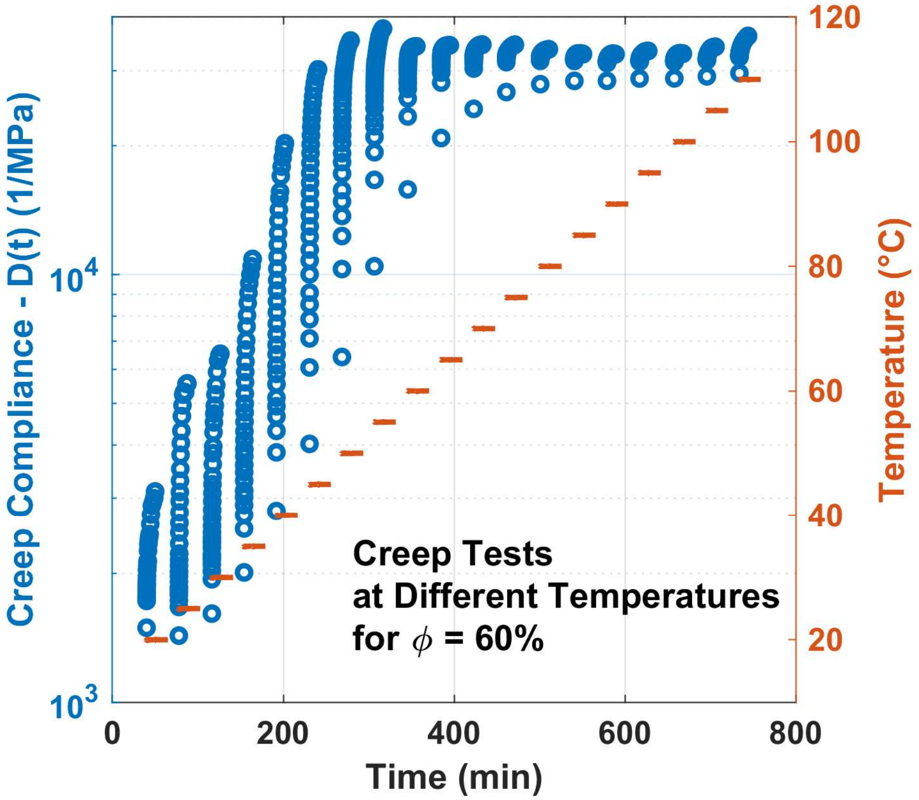

Figure S27. The creep test results performed at different temperatures at 5 °C for the TTS analysis. At each temperature, specimens were stretched with constant stress for 10 minutes, and their strain was measured over time. Similar to the relaxation tests, samples were then unloaded for 15 min to recover before the next cycle. The figure above shows the results for the syntactic foam with 60% HGMs per volume.

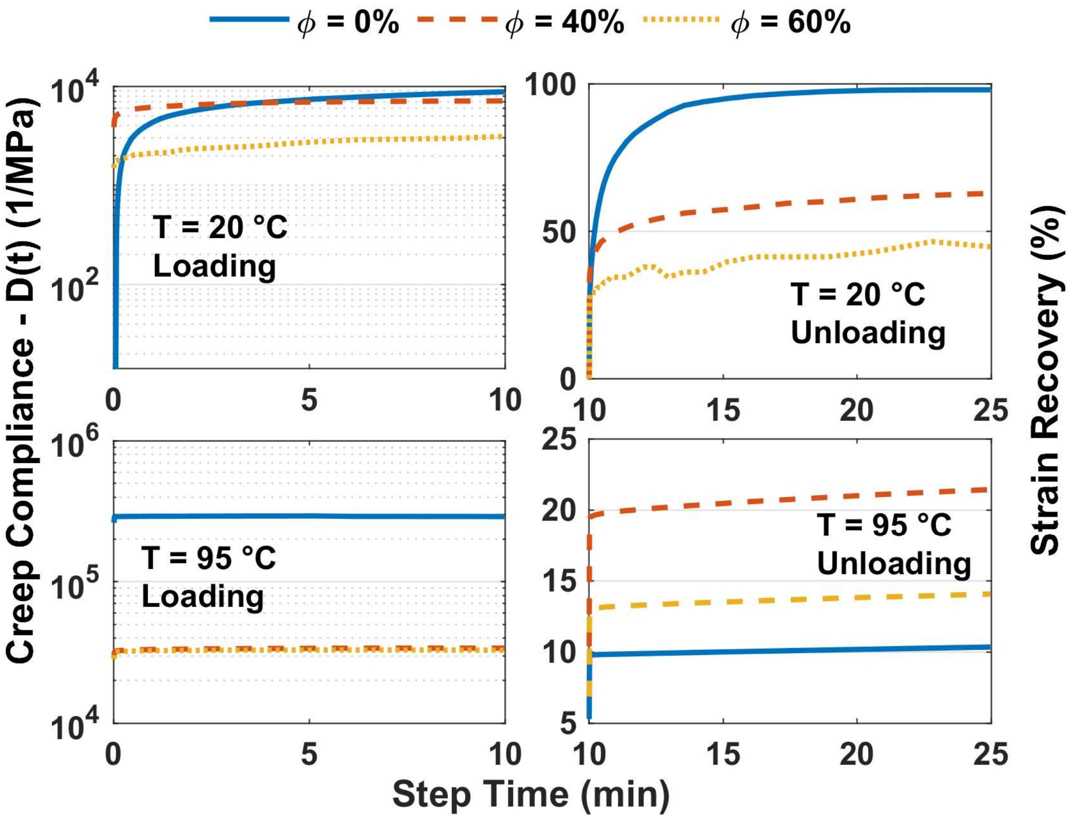

Figure S28. Comparison of the behavior of three samples with HGM volume fractions of 0, 40, and 60% in the creep test. The two temperatures of 20 and 95 °C are selected for this comparison to ensure the vitrimer matrix is in either a frozen or rubbery phase. The two plots on the left show the creep compliance during loading, and the two on the right show the recovery during unloading.

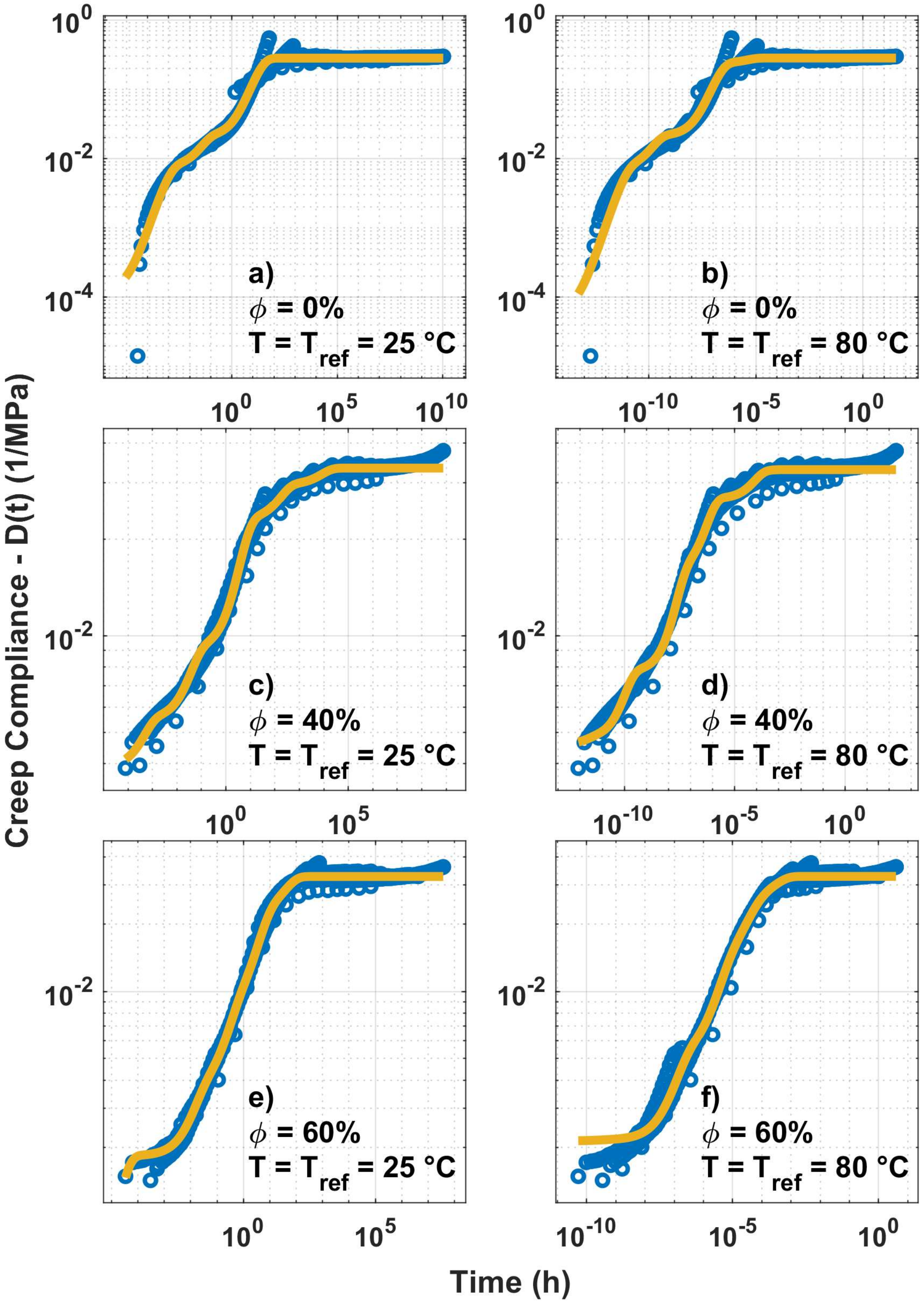

Figure S29. The generated master curves using the creep test data for samples with Φ = 0, 40, and 60% at 25 and 80 °C. The calculated points are shown with blue circles, and the discrete creep spectrum models fitted on those points are marked in yellow.

Table S9. The creep spectrum responses for samples with Φ = 0, 40, and 60% at 25 and 80 °C. These responses are calculated by fitting the model on the generated master curves (c.f., Figure S29). D_0 and η_0 are the initial response and viscosity, respectively. $D_{t\rightarrow\infty}$ is the equilibrium response.

T (°C)	25			80		
Φ (%)	0	40	60	0	40	60
D_0 (1/MPa)	1.17E-04	3.89E-03	1.65E-07	5.61E-05	4.57E-03	2.14E-03
$\boldsymbol{\eta_0}$ (MPa.s)	1.63E+15	4.69E+14	3.42E+13	3.35E+06	1.12E+08	3.57E+06
D_1 (1/MPa)	0.25973	3.77E-03	1.02E-02	0.21655	5.37E-04	7.59E-03
$\boldsymbol{\tau_1}$ (s)	7.32E+04	3.12E+07	1.58E+05	7.06E-04	9.78E-01	1.64E-02
D_2 (1/MPa)	1.30E-02	6.58E-03	1.45E-02	1.40E-02	8.12E-03	2.92E-03
$\boldsymbol{\tau_2}$ (s)	1.81E+02	6.82E+05	1.80E+04	7.75E-07	1.01E-04	5.48E-04
D_3 (1/MPa)	5.72E-08	1.40E-02	4.38E-03	7.34E-03	2.94E-03	2.14E-05
$\boldsymbol{\tau_3}$ (s)	2.615	17490	1299	2.639E-08	4.359E-07	9.837E-07
D_4 (1/MPa)	5.04E-04	3.55E-03	1.84E-03	1.18E-02	2.35E-04	6.85E-03
$\boldsymbol{\tau_4}$ (s)	1.797	154.9	0.07661	0.01521	7.444E-09	1.127
D_5 (1/MPa)	6.69E-03	3.44E-08	1.97E-05	2.40E-02	1.10E-02	7.41E-03
$\boldsymbol{\tau_5}$ (s)	3.48	11.74	32.95	1.78E-02	2.35E-03	0.21
D_6 (1/MPa)	4.42E-04	1.62E-03	1.71E-03	7.67E-03	5.66E-03	5.73E-03
$\boldsymbol{\tau_6}$ (s)	6.249	1.881	65.03	1.14E-02	0.2984	9.30E-02
$D_{t\rightarrow\infty}$ (1/MPa)	0.2807	2.95E-02	3.26E-02	0.2814	2.85E-02	3.05E-02

Table S10. Mechanical properties of the prepared specimens using different fraction ratios of HGMs using the tensile test results. Here, engineering stress and strain are used. The calculated modulus is at ϵ_{eng}= 0.2%.

Volume Fraction Φ (%)	Strength - $\boldsymbol{\sigma_{max}}$ (MPa)	Elongation at Break (%)	Modulus (MPa)	Elongation at $\boldsymbol{\sigma_{max}}$ (%)
0	27.78	15.91	1.36	4.00
40	17.37	4.39	0.78	4.38
50	12.76	2.62	0.63	2.61
60	10.63	1.91	0.72	1.90
70	2.11	1.87	0.20	1.62

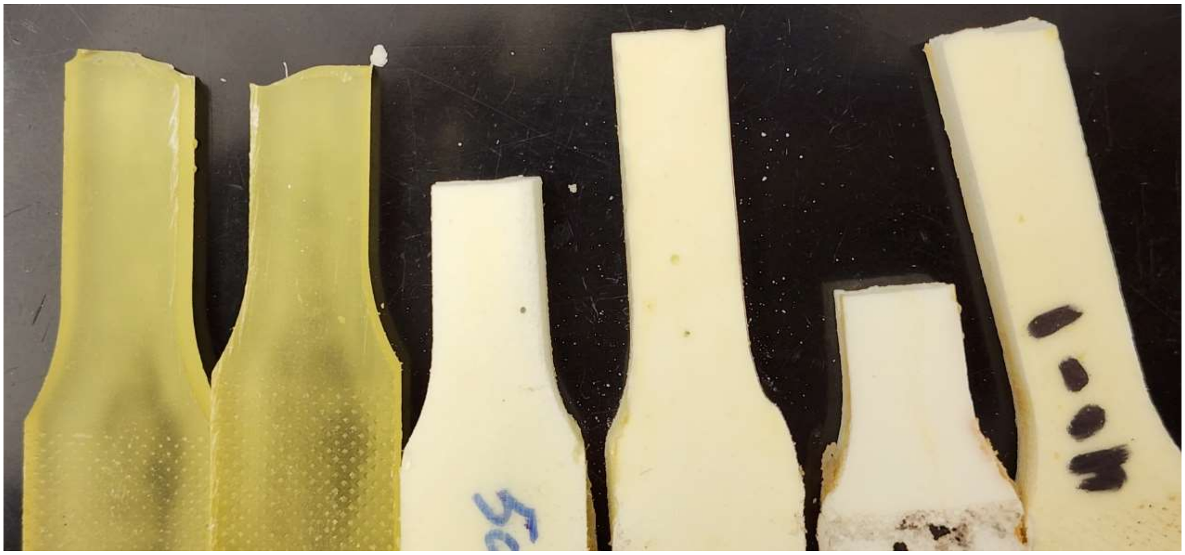

Figure S30. The fracture surfaces of the syntactic foam specimens (in white) compared to the pure SMV (colored dark yellow on the left).

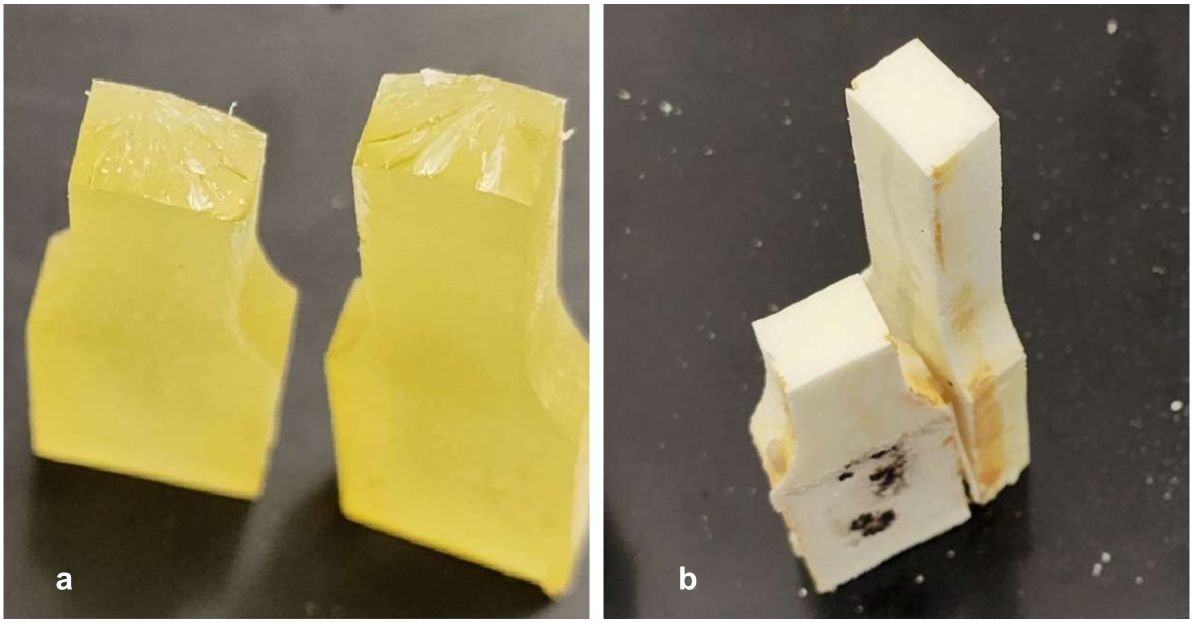

Figure S31. The typical fracture surface of a) pristine SMV, and b) syntactic foam due to tensile loading

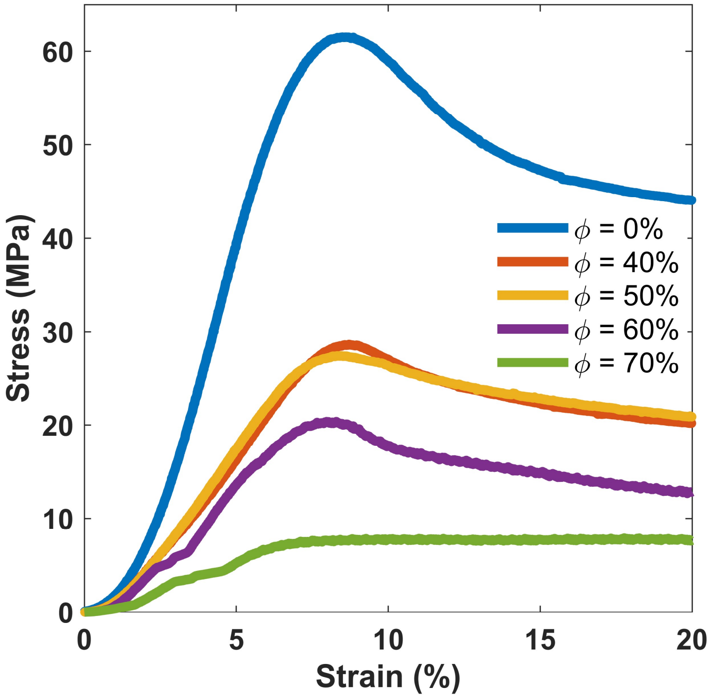

Figure S32. The true stress vs. true strain behavior of the prepared samples with different volume fractions of HGMs plotted only for the strains up to 20%.

Table S11. Mechanical properties of the prepared specimens using different fraction ratios of HGMs using the results of the compression test at room temperature. As for the tensile test, engineering stress and strain are used here. The calculated modulus is at ϵ_{eng}= 2%.

Volume Fraction Φ (%)	Strength $\boldsymbol{\sigma_{max}}$ (MPa)	Modulus E (MPa)	Strain at $\boldsymbol{\sigma_{max}}$ (%)
0	67.18	725	8.47
40	31.29	345.02	8.36
50	29.54	250.68	9.73
60	22.18	230.6	7.96
70	9.57	128.58	18.26

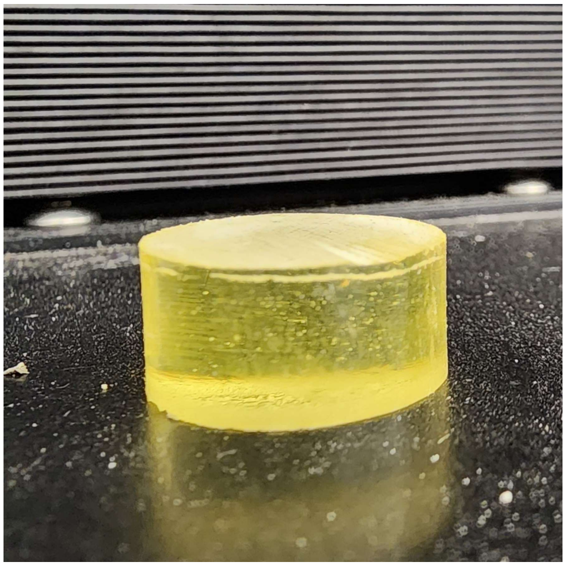

Figure S33. The pristine SMV recovered after the compression test at room temperature. No fractures or cracks are evident in the specimen.

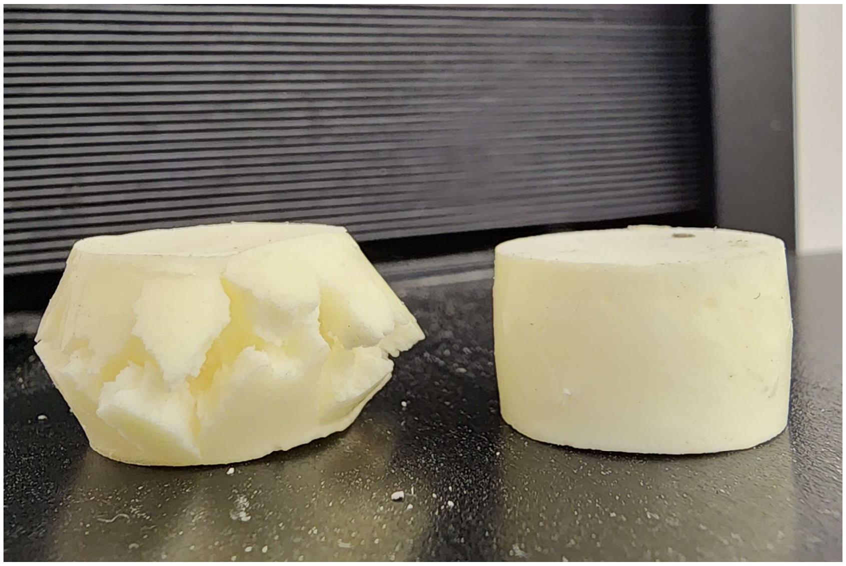

Figure S34. The damage experienced by the syntactic foams after the compression test at room temperature was proportional to their HGMs volume fraction. The higher the Φ was, the larger damage could be seen under the same strain. Note that the created cracks were at an angle almost equal to 45 °.

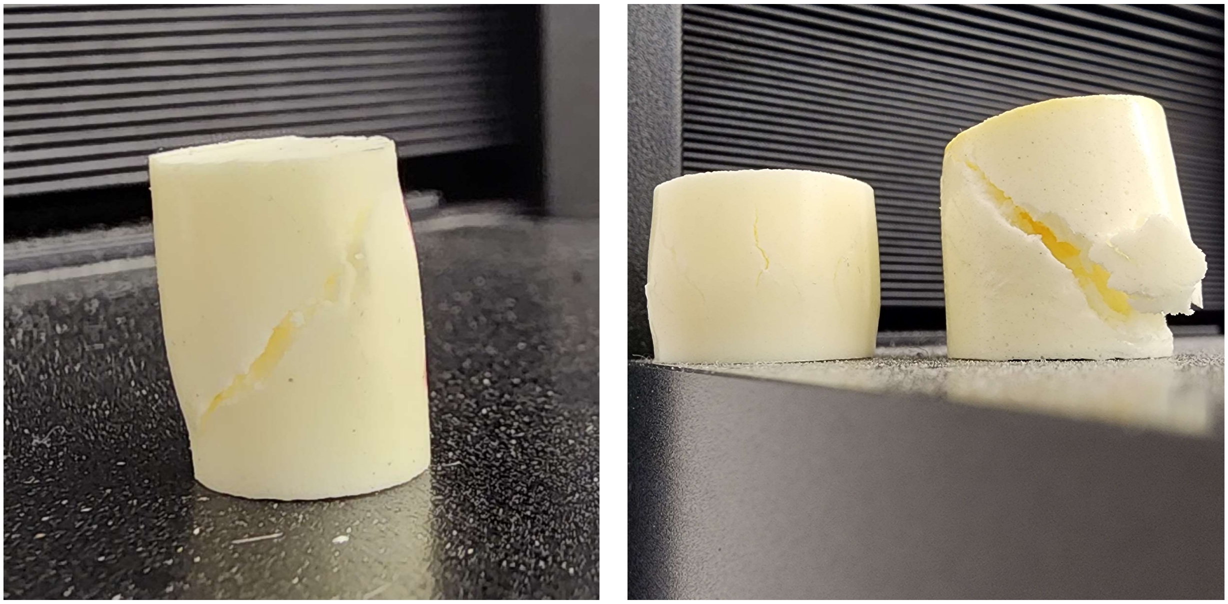

Figure S35. The two types of failure in the syntactic foams under compression at elevated temperature. While failure due to multiple fractures on the surface could still be seen, most specimens broke due to the propagation of a large diagonal crack.

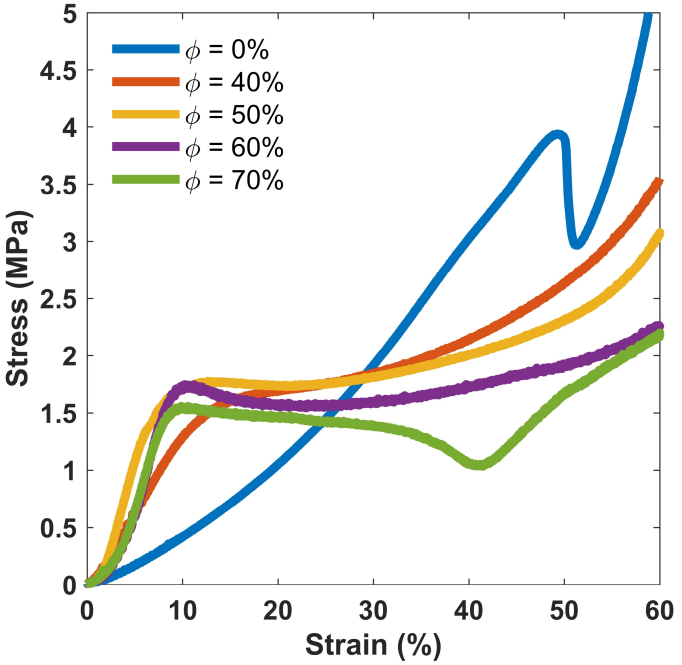

Figure S36. Engineering stress vs. strain for the compression test of samples with different volume fractions of HGMs at T = 60 °C.

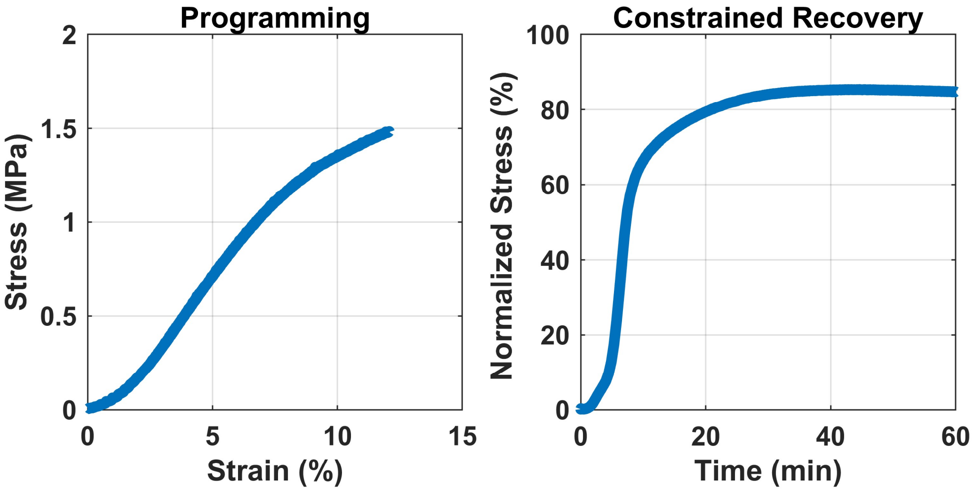

Figure S37. Programming syntactic foam with Φ = 50% before load reached the peak, resulting in stress recovery with high efficiency.

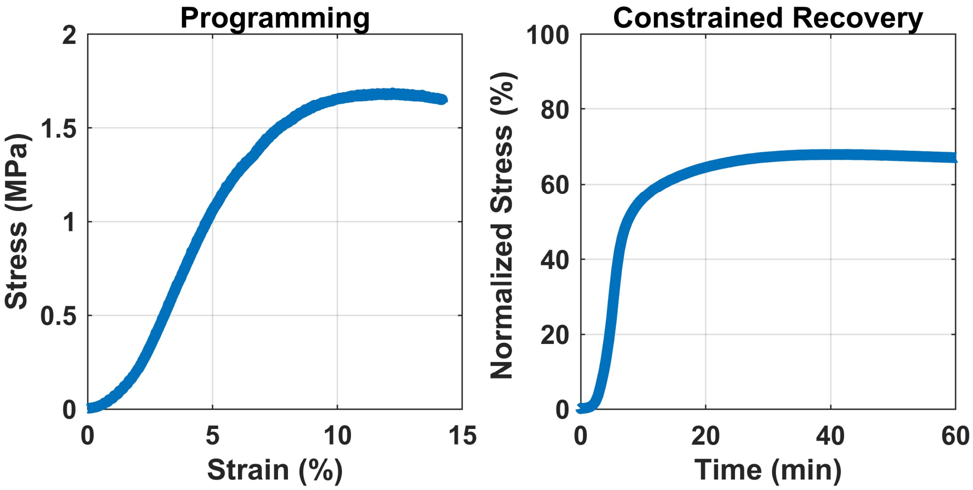

Figure S38. Programming syntactic foam with Φ = 50% past the peak in stress, resulting in a drop in the efficiency of stress recovery.

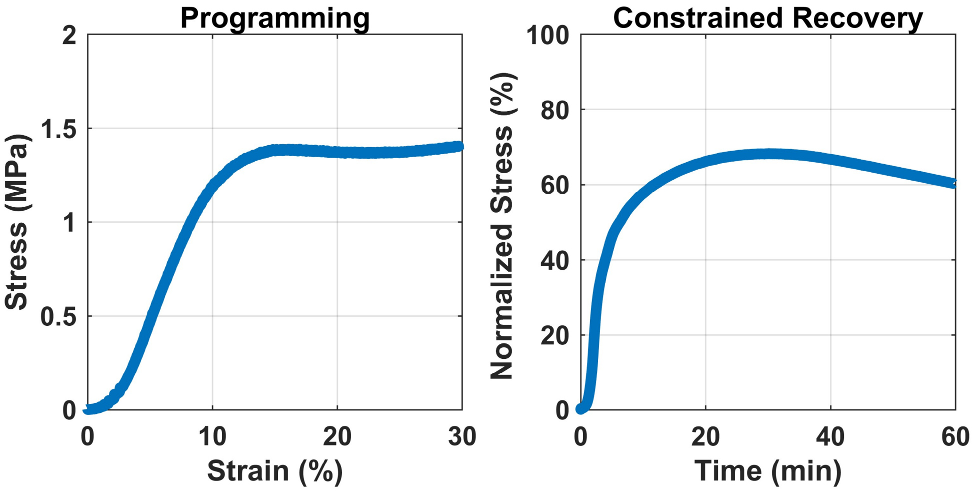

Figure S39. Continuing the programming of syntactic foam with Φ = 50% may lead to increased stress recovery efficiency if the slope of the stress-strain curve becomes positive again.

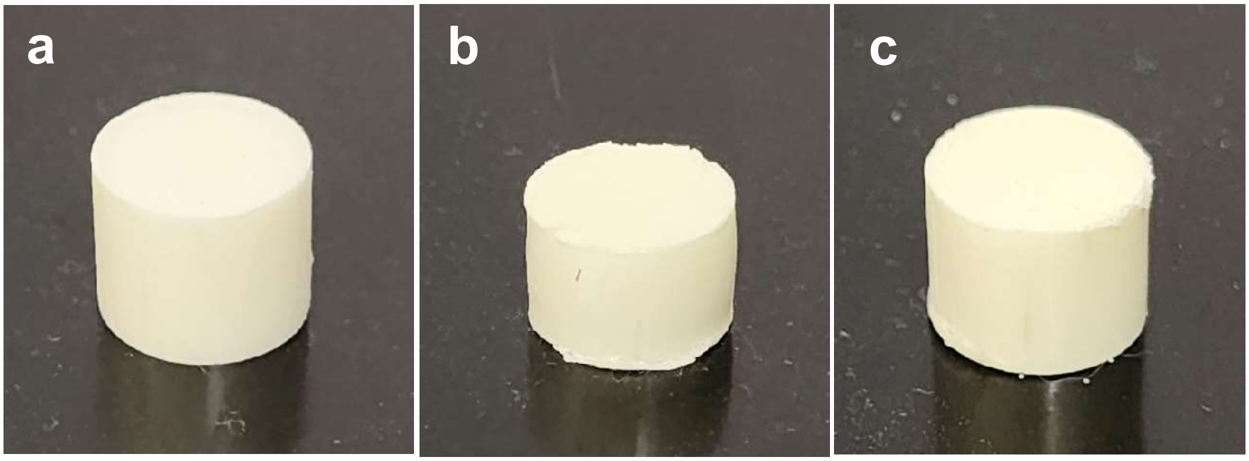

Figure S40. A cylindrical specimen during the programming and recovery processs: a) original shape, b) after programming, and c) after recovery.

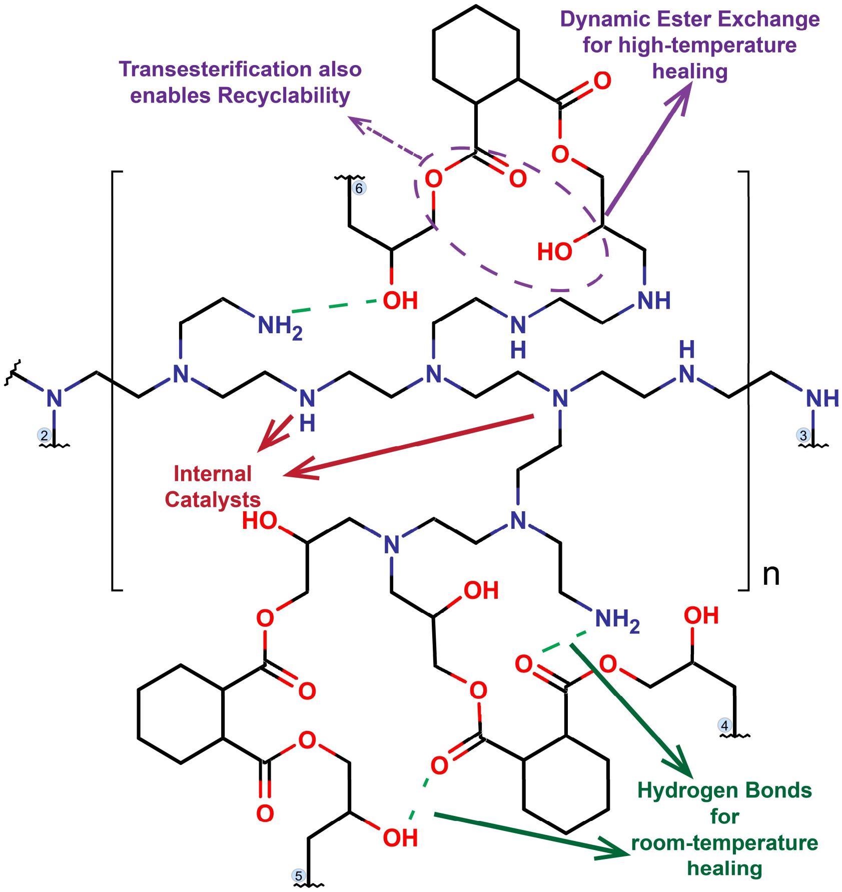

Figure S41. The chemical structure of the SMV.

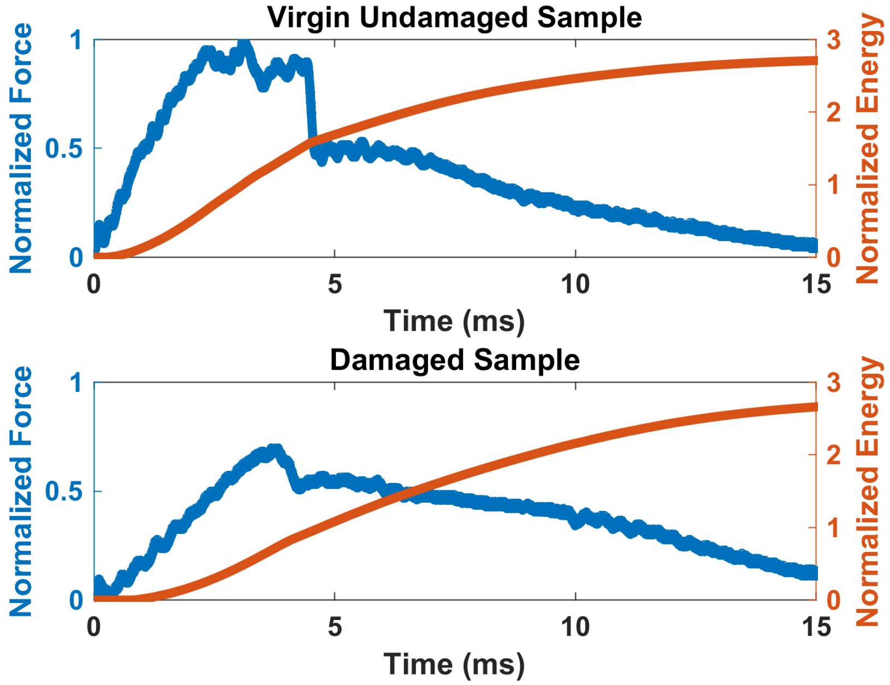

Figure S42. Change in the impact test results for the undamaged and the damaged samples at room temperature. Force and energy values are normalized by the maximum force (F_{max}) and the energy at the maximum force (E_{Fmax}) of the undamaged sample, respectively.

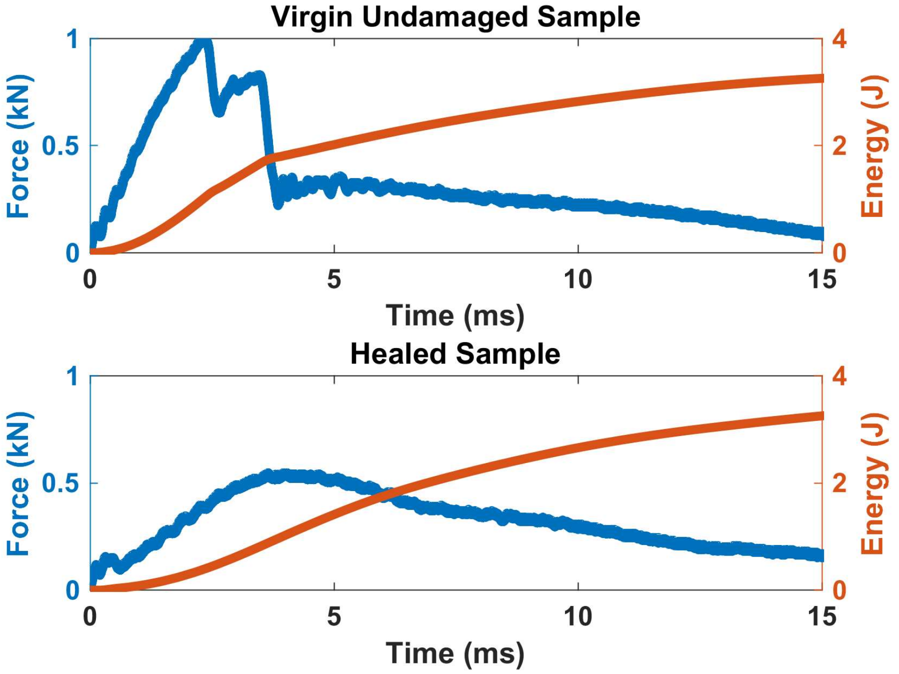

Figure S43. Change in the impact test results for the undamaged and the healed samples at room temperature. Force and energy values are normalized by the maximum force (F_{max}) and the energy at the maximum force (E_{Fmax}) of the undamaged sample, respectively.

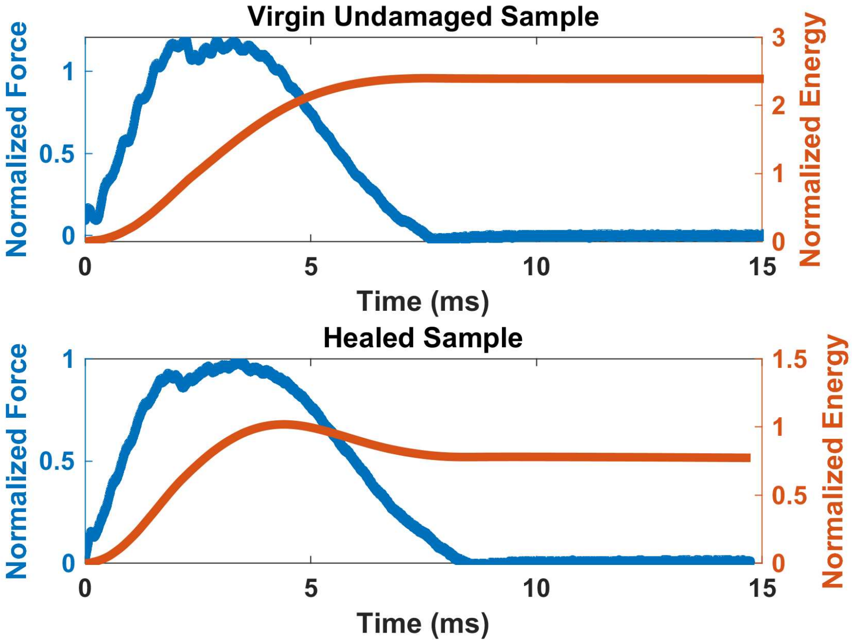

Figure S44. Change in the impact test results for the undamaged and the healed sample at high temperature (150 °C). Force and energy values are normalized by the maximum force (F_{max}) and the energy at the maximum force (E_{Fmax}) of the undamaged sample, respectively.

Since one of the healing mechanisms is activated at the high temperature of 150 °C, which is also the curing temperature of the foam, the improvement in the mechanical properties of a damaged specimen may partly be due to the polymer's post-curing. Since a longer curing time may lead to the formation of additional crosslinks, it may be speculated that the potential increase in the stiffness of the polymer was wrongly considered as healing. To examine this, the DMA tests were repeated by increasing the temperature to 150 °C. Once reached, samples were kept at this condition for 1 h, and their rheological properties were recorded. As Figure S.35 indicates, the storage and loss moduli only slightly fluctuated over this period. The storage modulus, in particular, decreased for the syntactic foam samples rather than increased. Therefore, the better mechanical properties after the high-temperature healing cannot be attributed to post-curing or increased crosslink density.

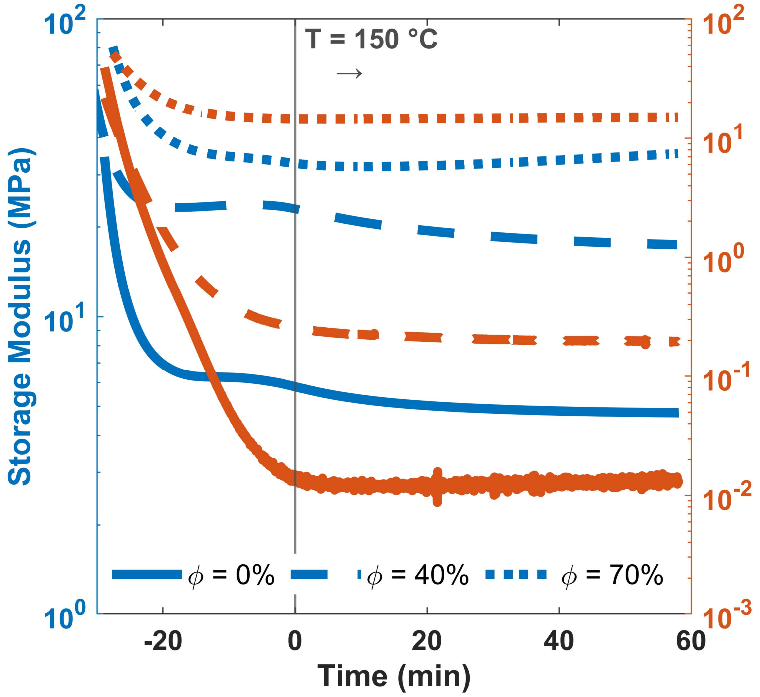

Figure S45. The change in storage and loss moduli of the pure SMV and two syntactic foams with the lowest and highest volume fractions as the temperature was increased and then maintained at 150 °C for 1 h.

S.1. Further Discussion Regarding the Theoretical and Practical Limits for Volume Fraction of HGMs

The theoretical limit on the volume fraction of particulates in a composite is critical for two reasons: modeling and manufacturing limitations.

Theoretically, the lower limit on particle volume fraction is the inclusion of only one particle in an arbitrary unit volume of the matrix. In practice, only a few particles bear no significance on any of the physical or chemical properties of the obtained material. Moreover, incorporating only a few particles in a matrix cannot be considered a composite, and those inclusions should be treated as impurities or contaminants. Nevertheless, the lower limit on the inclusion volume fraction is usually not challenging as lower volumes can be easily incorporated and distributed uniformly in a matrix. However, the higher possible limitation on the volume fraction is quite a tricky question from the manufacturing standpoint.

From a modeling perspective, many analytical models are based on the assumption of a representative volume element (RVE) around inclusions in which the matrix is assumed to be infinitely large. This assumption is principally equivalent to a large enough matrix for which the effect of neighboring inclusions can be reasonably ignored [1]. As a result, the model performs well only when the volume fraction is relatively low [2]. The error of the model grows with the inclusions' volume fraction. Many models suggest a limit until which they deem their model's prediction acceptable. These models become inaccurate at higher volume fractions, which may apply to some syntactic foams. As the mechanical model of these volume fractions is too complicated to solve explicitly, engineers usually use other methods, such as the finite element method, to numerically calculate the mechanical behavior of such structures [3].

From the manufacturing point of view, the question of the maximum volume fraction attainable in a syntactic foam is also very challenging. Historically, tiling areas and packing volumes of different shapes with different geometries have interested mathematicians in optimization and geometry fields [4].

Assuming the spherical hollow glass bubbles in a syntactic foam as monodispersed solid spheres, their maximum number that can fit in a shape memory polymer matrix can be estimated by the number of spheres that can fit inside a spherical or cubic unit volume. These problems are usually solved numerically for different numbers of spheres [5]. The maximum volume fraction of spheres packed densely in spherical unit cells has been calculated numerically for *many* particles and has so far reached a maximum density of approximately 55.78% for 61 spheres [6]. The SMP matrix can also be considered an open volume of Euclidean space. It is proved that a periodic face-centered cubic (FCC) or a hexagonal closed-pack (HCP) lattice with a packing density of 74.5%, which Kepler initially hypothesized, has the maximum packing efficiency in a volume [7]. However, this maximum limit is only attainable through the crystallization process, in which all particles are perfectly placed beside each other without displacement or dislocations. This process is practically impossible to accomplish while preparing syntactic foams. The mixing process during syntactic foam’s preparation is probably better replicated in the random close packing problems. The lowest density for randomly packed spheres is between 51% and 59% based on the packing conditions [8]. When spheres are poured into a container, the volume fraction can reach 62.5% [9]. Shaking the bubbles until they settle to the densest packing order enables a volume fraction of up to 64.5% [10]. It is important to note that these problems are usually empirically or simulated numerically. Therefore, the randomness of the simulation is slightly challenging to define and implement.

Ideally, each sphere in an SMP-based syntactic foam is surrounded by at least a thin layer of polymer. In other words, no two bubbles touch each other directly. Otherwise, the elasticity of the particles translates even minuscule deformations throughout the material, superseding the viscoelastic-viscoplastic behavior of the matrix. Here, the material will not be able to accommodate a shape change plastically unless the bubbles are damaged, or a series of them switch their positions simultaneously. The minimum thickness between neighboring spheres is dependent on the material type. For polymeric syntactic foam, this layer depends on the type and size of the polymer chain and the crosslinking between them and, therefore, is probably too complicated to determine. The conformity of the syntactic foam also depends on the interconnectivity in the polymer matrix. The higher the interconnectivity inside the lattice, the easier the viscoelastic polymeric network can conform to the applied load. This connectivity is studied by the percolation threshold and is out of the scope of this study [11]. However, it is mentioned that if the volume fraction of the polymers falls below 33%, it can no longer enfold all bubbles in a monodispersed mixture [12]. The existence of such a layer, with any thickness, obviously reduces the volume fraction from the theoretical packing density limit.

It should be noted that although the added bubbles in making a syntactic foam are spherical, it is theoretically possible that they slightly deform their shape by pressure to fit them into a denser formation. Therefore, it is essential to consider a packing problem for ellipsoidal shapes, which may better represent the bubbles in some conditions. Any ellipsoid has a higher packing density than a spherical packing. To date, the two densest ellipsoidal structures are simple monoclinic crystals with two ellipsoids of different orientations with about 77% density[13] and quasi-square-triangular crystal containing 24 ellipsoids in a repeated unit cell with slightly higher densities[14]. However, it should also be noted that unless the prepared syntactic foam is kept contained, the

induced strain energy in the bubbles makes them tend naturally to reach their more stable spherical shapes, bouncing out of their ellipsoidal shapes. Therefore, there is only a low probability of predominantly observing this packing pattern in a foam.

Another assumption regarding the bubbles in a syntactic foam was that they were all the same size (i.e., monodispersed as compared to polydispersed). While theoretically, it may be possible to collect bubbles of just one size through complicated series of filtrations, it is almost impossible to manufacture single-sized bubbles in large quantities. In particular, the hollow glass microbubbles used in preparing syntactic foams are always made with a quasi-normal distribution of different diameters. Along with their density and other mechanical properties, the particles are usually marketed with their median diameter, and at times, with some hints at the distribution of different sizes in the product. For example, for the K15 HGMs used in this study, 3M advertises their distribution as 30 μm, 65 μm, and 105 μm for the first 10th percentile, 50th percentile, and 90th percentile by volume, respectively. The effective top size of this type of bubble is reported to be 115 μm [15].

The fact that all HGMs used in making syntactic foams are not of the same size creates further challenges in solving the close-packing problem, for which, to the best of the authors' knowledge, there is no verified solution to this date. Theoretically, the packing density may increase by the proper gradation of particle size. For example, for a binary system, if one sphere has a diameter 0.414 of the other one, it can theoretically fit perfectly in the voids between the closed-packed FCC structure. If this trend could have been continued for n times, the packing density, D, would have been calculated by

$$D = 1 - 0.255^n, \quad (1)$$

which theoretically equals 1 as n approaches infinity.

Other studies examined different ratios [16]. It is shown that the packing density for any packing structure increases as long as the diameter ratio is around 0.29 [17]. However, for binary systems of proportions up to approximately 0.66, there are packing structures surpassing the FCC lattice's density [18]. Calculations of such to selectively choose different particles size are commonly used for concrete aggregate gradation research [19], with aggregates diameter changing from more than an inch to the size of a grain of sand. The volume fraction of the materials smaller than size d, f_d, can be calculated [20] by

$$f_d = \left(\frac{d}{D}\right)^n \tag{2}$$

where D is the size of the largest particle in the mixture. The maximum density is obtained when n equals 0.5. However, n is usually considered 0.45 in practice [21].

Compared to these mixtures made from calculated amounts of selected discrete sizes, a continuous distribution with a comparatively much smaller standard deviation that applies to the HGMs does not increase the packing density but will probably lower it. Unfortunately, calculating the theoretical limit for this situation is not available to the best of the authors' knowledge. Currently, the research is limited to how the ratio between the diameter of two or three spheres affects the volume fraction. However, studies have simulated the polydispersed hard spheres with a size distribution [22].

It must be noted that creating very tiny spaces due to a perfect gradation of the particles may also affect the amount of polymer matrix that could penetrate those spaces. Potentially it may not be favorable energetically for the polymer matrix to take the shape of those small spaces, which leads to the creation of voids and may reduce the volume fraction of the hollow bubbles with respect to the whole mixture.

It must also be noted that the manufacturing of syntactic foams is always marred with the loss of many glass bubbles due to breakage. This breakage may happen at any time during the manufacturing of the glass bubbles, their handling and transportation, preparation of the syntactic foam, especially mixing, molding, or extrusion, and even due to thermal stresses during curing. Due to its additional complexity, this critical phenomenon is disregarded here. It is assumed the syntactic foam is made with undamaged spheres. The amount of created voids during manufacturing is also considered to be negligible. In addition, since the viscosity of the mixture increases with an increase in the bubbles' volume fraction, the mixing process becomes quite complicated. Therefore, it should be noted that reaching the higher limits may, in practice, increase the chance of crushing the bubbles or creating voids in the foam. Also, obtaining a perfectly uniform mixture is quite challenging. However, it becomes even more difficult as the polymer percentage decreases, and the above increases the challenge even more.

Supplemental References

(1) Christensen, R. M.; Lo, K. H. Solutions for effective shear properties in three phase sphere and cylinder models. *Journal of the Mechanics and Physics of Solids* **1979**, *27* (4), 315-330. DOI: https://doi.org/10.1016/0022-5096(79)90032-2. Li, G.; Zhao, Y.; Pang, S.-S. Four-phase sphere modeling of effective bulk modulus of concrete. *Cement and Concrete Research* **1999**, *29* (6), 839-845. DOI: https://doi.org/10.1016/S0008-8846(99)00040-X. Li, G.; Zhao, Y.; Pang, S.-S.; Li, Y. Effective Young's modulus estimation of concrete. *Cement and Concrete Research* **1999**, *29* (9), 1455-1462. DOI: https://doi.org/10.1016/S0008-8846(99)00119-2. Goodier, J. Concentration of stress around spherical and cylindrical inclusions and flaws. *TASME* **1933**, *55*, 39. Hashin, Z. The Elastic Moduli of Heterogeneous Materials. *Journal of Applied Mechanics* **1962**, *29* (1), 143-150. DOI: 10.1115/1.3636446. Lee, K. Y.; Paul, D. R. A model for composites containing three-

dimensional ellipsoidal inclusions. *Polymer* **2005**, *46* (21), 9064-9080. DOI: https://doi.org/10.1016/j.polymer.2005.06.113. Saadat, F.; Birman, V.; Thomopoulos, S.; Genin, G. M. Effective elastic properties of a composite containing multiple types of anisotropic ellipsoidal inclusions, with application to the attachment of tendon to bone. *Journal of the Mechanics and Physics of Solids* **2015**, *82*, 367-377. DOI: https://doi.org/10.1016/j.jmps.2015.05.017 From NLM PubMed-not-MEDLINE. Progelhof, R.; Throne, J.; Ruetsch, R. Methods for predicting the thermal conductivity of composite systems: a review. *Polymer Engineering & Science* **1976**, *16* (9), 615-625.

(2) Gu, J.; Sun, H.; Fang, C. A multi-branch finite deformation constitutive model for a shape memory polymer based syntactic foam. *Smart Materials and Structures* **2015**, *24* (2), 025011. DOI: 10.1088/0964-1726/24/2/025011.

(3) Yan, C.; Li, G. Design oriented constitutive modeling of amorphous shape memory polymers and Its application to multiple length scale lattice structures. *Smart Materials and Structures* **2019**, *28* (9), 095030. DOI: 10.1088/1361-665X/ab230c.

(4) Lodi, A.; Martello, S.; Monaci, M. Two-dimensional packing problems: A survey. *European Journal of Operational Research* **2002**, *141* (2), 241-252. DOI: https://doi.org/10.1016/S0377-2217(02)00123-6. Chernov, N.; Stoyan, Y.; Romanova, T. Mathematical model and efficient algorithms for object packing problem. *Computational Geometry* **2010**, *43* (5), 535-553. DOI: https://doi.org/10.1016/j.comgeo.2009.12.003.

(5) Gensane, T. Dense packings of equal spheres in a cube. *the electronic journal of combinatorics* **2004**, R33-R33.

(6) Huang, W.; Yu, L. Serial symmetrical relocation algorithm for the equal sphere packing problem. *arXiv preprint arXiv:1202.4149* **2012**. Pfoertner, H. Numerical results for densest packing of n equal spheres in a larger sphere with radius= 1. In *The Online Encyclopedia of Integer Sequences*, 2013.

(7) Hales, T.; Adams, M.; Bauer, G.; Dang, T. D.; Harrison, J.; Hoang, L. T.; Kaliszyk, C.; Magron, V.; McLaughlin, S.; Nguyen, Tat T.; et al. A FORMAL PROOF OF THE KEPLER CONJECTURE. *Forum of Mathematics, Pi* **2017**, *5*, e2. DOI: 10.1017/fmp.2017.1 From Cambridge University Press Cambridge Core. Gauss, C. Untersuchungen uber die Eigenschaften der positiven ternaren quadratishen Former von Ludwig August Seeber. *Gottingische gelehrte Anzeigen* **1831**.

(8) Dong, K.; Yang, R.; Zou, R.; Yu, A. Role of interparticle forces in the formation of random loose packing. *Physical review letters* **2006**, *96* (14), 145505. DOI: 10.1103/PhysRevLett.96.145505 From NLM PubMed-not-MEDLINE. Pusey, P. N.; van Megen, W. Observation of a glass transition in suspensions of spherical colloidal particles. *Physical review letters* **1987**, *59* (18), 2083. DOI: 10.1103/PhysRevLett.59.2083 From NLM Publisher.

(9) McGeary, R. Mechanical packing of spherical particles. *Journal of the American ceramic Society* **1961**, *44* (10), 513-522.

(10) Torquato, S.; Truskett, T. M.; Debenedetti, P. G. Is Random Close Packing of Spheres Well Defined? *Physical Review Letters* **2000**, *84* (10), 2064-2067. DOI: 10.1103/PhysRevLett.84.2064 From NLM Publisher.

(11) van der Marck, S. C. Percolation thresholds of the duals of the face-centered-cubic, hexagonal-close-packed, and diamond lattices. *Physical Review E* **1997**, *55* (6), 6593-6597. DOI: 10.1103/PhysRevE.55.6593. Dalton, N. W.; Domb, C.; Sykes, M. F. Dependence of critical concentration of a dilute ferromagnet on the range of interaction. *Proceedings of the Physical Society* **1964**, *83* (3), 496. DOI: 10.1088/0370-1328/83/3/118. Hu, Y.; Charbonneau, P. Percolation thresholds on high-dimensional Dn and E8-related lattices. *Physical Review E* **2021**, *103* (6), 062115. DOI: 10.1103/PhysRevE.103.062115 From NLM PubMed-not-MEDLINE. Xu, F.; Xu, Z.; Yakobson, B. I. Site-percolation threshold of carbon nanotube fibers—Fast inspection of percolation with Markov stochastic theory. *Physica A: Statistical Mechanics and its Applications* **2014**, *407*, 341-349. DOI: https://doi.org/10.1016/j.physa.2014.04.013. Xun, Z.; Hao, D.; Ziff, R. M. Site and bond percolation thresholds on regular lattices with compact extended-range neighborhoods in two and three dimensions. *Physical Review E* **2022**, *105* (2), 024105. DOI: 10.1103/PhysRevE.105.024105 From NLM PubMed-not-MEDLINE. Gawron, T.; Cieplak, M. Site percolation thresholds of fcc lattice. *Acta Phys. Pol. A* **1991**, *80* (3), 461-464. Horton, M. K.; Moram, M. A. Alloy composition fluctuations and percolation in semiconductor alloy quantum wells. *Applied Physics Letters* **2017**, *110* (16), 162103. DOI: 10.1063/1.4980089. Sykes, M. F.; Essam, J. W. Critical Percolation Probabilities by Series Methods. *Physical Review* **1964**, *133* (1A), A310-A315. DOI: 10.1103/PhysRev.133.A310.

(12) Shutov, F. A. Syntactic polymer foams. In *Chromatography/foams/copolymers*, Springer, 1986; pp 63-123.

(13) Donev, A.; Stillinger, F. H.; Chaikin, P. M.; Torquato, S. Unusually Dense Crystal Packings of Ellipsoids. *Physical Review Letters* **2004**, *92* (25), 255506. DOI: 10.1103/PhysRevLett.92.255506 From NLM PubMed-not-MEDLINE.

(14) Jin, W.; Jiao, Y.; Liu, L.; Yuan, Y.; Li, S. Dense crystalline packings of ellipsoids. *Physical Review E* **2017**, *95* (3), 033003. DOI: 10.1103/PhysRevE.95.033003 From NLM PubMed-not-MEDLINE.

(15) Product Information. In *3M™ Glass Bubbles: K Series, S Series and iM Series*, 3M, Ed.; St. Paul, MN, 2013.

(16) Schurmann, A.; Vallentin, F. Computational Approaches to Lattice Packing and Covering Problems. *Discrete & Computational Geometry* **2006**, *35* (1), 73-116. DOI: 10.1007/s00454-005-1202-2.

(17) Zong, C. From deep holes to free planes. *Bulletin of the American Mathematical Society* **2002**, *39* (4), 533-555.

(18) O'Toole, P. I.; Hudson, T. S. New High-Density Packings of Similarly Sized Binary Spheres. *The Journal of Physical Chemistry C* **2011**, *115* (39), 19037-19040. DOI: 10.1021/jp206115p.

(19) Richardson, D. N. Aggregate Gradation Optimization--Literature Search. **2005**.

(20) Fuller, W. B.; Thompson, S. E. The Laws of Proportioning Concrete. *Transactions of the American Society of Civil Engineers* **1907**, *59* (2), 67-143. DOI: doi:10.1061/TACEAT.0001979.

(21) Shilstone, J. M.; Shilstone, J. Performance-based concrete mixtures and specifications for today. *Concrete international* **2002**, *24* (2), 80-83.

(22) Al-Raoush, R.; Alsaleh, M. Simulation of random packing of polydisperse particles. *Powder technology* **2007**, *176* (1), 47-55. Bolhuis, P. G.; Kofke, D. A. Monte Carlo study of freezing of polydisperse hard spheres. *Physical Review E* **1996**, *54* (1), 634. Corwin, E. I.; Clusel, M.; Siemens, A. O.; Brujić, J. Model for random packing of polydisperse frictionless spheres. *Soft Matter* **2010**, *6* (13), 2949-2959. Santiso, E.; Müller, E. A. Dense packing of binary and polydisperse hard spheres. *Molecular Physics* **2002**, *100* (15), 2461-2469. Schaertl, W.; Sillescu, H. Brownian dynamics of polydisperse colloidal hard spheres: Equilibrium structures and random close packings. *Journal of Statistical Physics* **1994**, *77* (5), 1007-1025. Yang, A.; Miller, C.; Turcoliver, L. Simulation of correlated and uncorrelated packing of random size spheres. *Physical review E* **1996**, *53* (2), 1516. DOI: 10.1103/physreve.53.1516 From NLM PubMed-not-MEDLINE.
